# Toll-like receptor-targeted anti-tumor therapies: Advances and challenges

**DOI:** 10.3389/fimmu.2022.1049340

**Published:** 2022-11-21

**Authors:** Yang Yang, Hongyi Li, Christina Fotopoulou, Paula Cunnea, Xia Zhao

**Affiliations:** ^1^ Development and Related Disease of Women and Children Key Laboratory of Sichuan Province, Key Laboratory of Birth Defects and Related Diseases of Women and Children, Ministry of Education, Department of Gynecology and Obstetrics, West China Second Hospital, Sichuan University, Chengdu, China; ^2^ Division of Cancer, Department of Surgery and Cancer, Faculty of Medicine, Imperial College London, London, United Kingdom

**Keywords:** toll-like receptors, cancer, targeted therapy, immunotherapy, clinical trials

## Abstract

Toll-like receptors (TLRs) are pattern recognition receptors, originally discovered to stimulate innate immune reactions against microbial infection. TLRs also play essential roles in bridging the innate and adaptive immune system, playing multiple roles in inflammation, autoimmune diseases, and cancer. Thanks to the immune stimulatory potential of TLRs, TLR-targeted strategies in cancer treatment have proved to be able to regulate the tumor microenvironment towards tumoricidal phenotypes. Quantities of pre-clinical studies and clinical trials using TLR-targeted strategies in treating cancer have been initiated, with some drugs already becoming part of standard care. Here we review the structure, ligand, signaling pathways, and expression of TLRs; we then provide an overview of the pre-clinical studies and an updated clinical trial watch targeting each TLR in cancer treatment; and finally, we discuss the challenges and prospects of TLR-targeted therapy.

## Introduction

Toll-like receptors (TLRs) are type I transmembrane glycoproteins (1) with evolutionarily conserved structures, existing in a wide variety of species from plants to vertebrates (2). Since the discovery in 1996 of Toll receptor protein that contributes to Drosophila’s anti-fungal response and embryonic development (3, 4), 13 functionally active homologs of Toll receptor-TLRs, have been identified in humans and mice, of which TLR1-9 and TLR11 are conserved in both species (5–7). However, TLR11 is non-functional in human, presented only by a pseudogene (8).TLR12 and 13 do not exist in humans, while TLR10 in mice is non-functional due to a retrovirus insertion (6).

Pattern recognition receptors (PRRs) are key components of innate immunity because of their ability to sense infection, elicit intracellular signaling cascades and initiate immune responses that ultimately eliminate pathogens and infected cells (9). As a group of important PRRs (3), TLRs recognize diverse microbial pathogens (e.g., lipids, peptides, carbohydrates, and nucleic acids) by their conserved molecular patterns, indicated as pathogen-associated molecular patterns (PAMPs), and initiate immune responses (10, 11). Almost all cells of the immune system (e.g., macrophages, B lymphocytes, dendritic cells, mast cells, neutrophils, etc.) as well as epithelial cells, endothelial cells, adipocytes, and cardiomyocytes recognize pathogens *via* TLRs (9). Recognition of microbial products by TLRs activates the innate immune response and triggers the activation of downstream signaling pathways in which myeloid differentiation factor 88 (MyD88) and toll-IL-1 receptor structural domain (TRIF) lead to the activation of NF-κB and subsequent transcription of pro-inflammatory cytokines including tumor necrosis factor-α (TNF-α), IL-1 and IL-6 (12). TLRs also recognize conserved molecular structures of host-derived molecules, often referred to as damage-associated molecular patterns (DAMPs) (13), derived after cell death and extracellular matrix (ECM) degradation from tissue damage caused by trauma or infection. TLRs have also been found to play critical roles in many other activities, including adaptive immune responses (14), differentiation and development (15, 16), tissue regeneration (17–19), cell cycle regulation (20, 21), and metabolism (22, 23).

As a group of regulators of various cellular functions, it is unsurprising that TLRs also exert their versatility in the process of carcinogenesis and tumor development, where their functions are augmented or dysregulated, resulting in either anti-tumor or pro-tumor responses (24–26). The exploitation of TLR anti-tumor activities has shown great promise in cancer immunotherapies, with some synthesized TLR agonists already approved by FDA for clinical use (27); however, problems still exist relating to their use, including limited translation rate from bench to bedside, possible immunosuppression induced by TLR agonists, and safety issues (28, 29).

In this article, we review the basic features of TLRs. We report on the applications of TLR-targeted treatment in anti-cancer therapies in clinical trials, encompassing various combinatory therapeutic strategies. Lastly, we review the challenges facing TLR-targeted therapy at present.

## TLR structures and ligands

### TLR structures

TLRs are membrane-spanning proteins (3) with an extracellular N-terminal domain consisting of 19-25 tandem leucine-rich repeat motifs (2), displaying a horseshoe tertiary structure (30, 31), which is responsible for ligand recognition (2). Next to the ectodomain is a transmembrane region (3), connecting the intracellular portion: the Toll/IL-1 receptor (TIR) domain in the C-terminal tail, which is homologous to IL-1 receptor family’s cytoplasmic region (32) and required for downstream signaling (10).

All the TLRs are synthesized in the endoplasmic reticulum (ER), then transferred to Golgi apparatus, and finally migrate to the plasma membrane or intracellular endosomes (33).

Human TLRs can be classified into two groups by sub-cellular localization: 1) the cell surface TLRs, TLR1, 2, 4, 5, 6, and 10, which reside on the plasma membrane (4), traveling to phagosomes when activated (34), 2) the intracellular TLRs, TLR7, 8, 9, which are expressed on endosomes inside the cells, with the LRR domain facing the inner cavity of the compartment, sampling their ligands (31). TLR3 are also primarily expressed on endosomes, yet it has been reported to express on the cell surface of fibroblasts, epithelial cells, and macrophages as well (35, 36).

**Figure 1:**
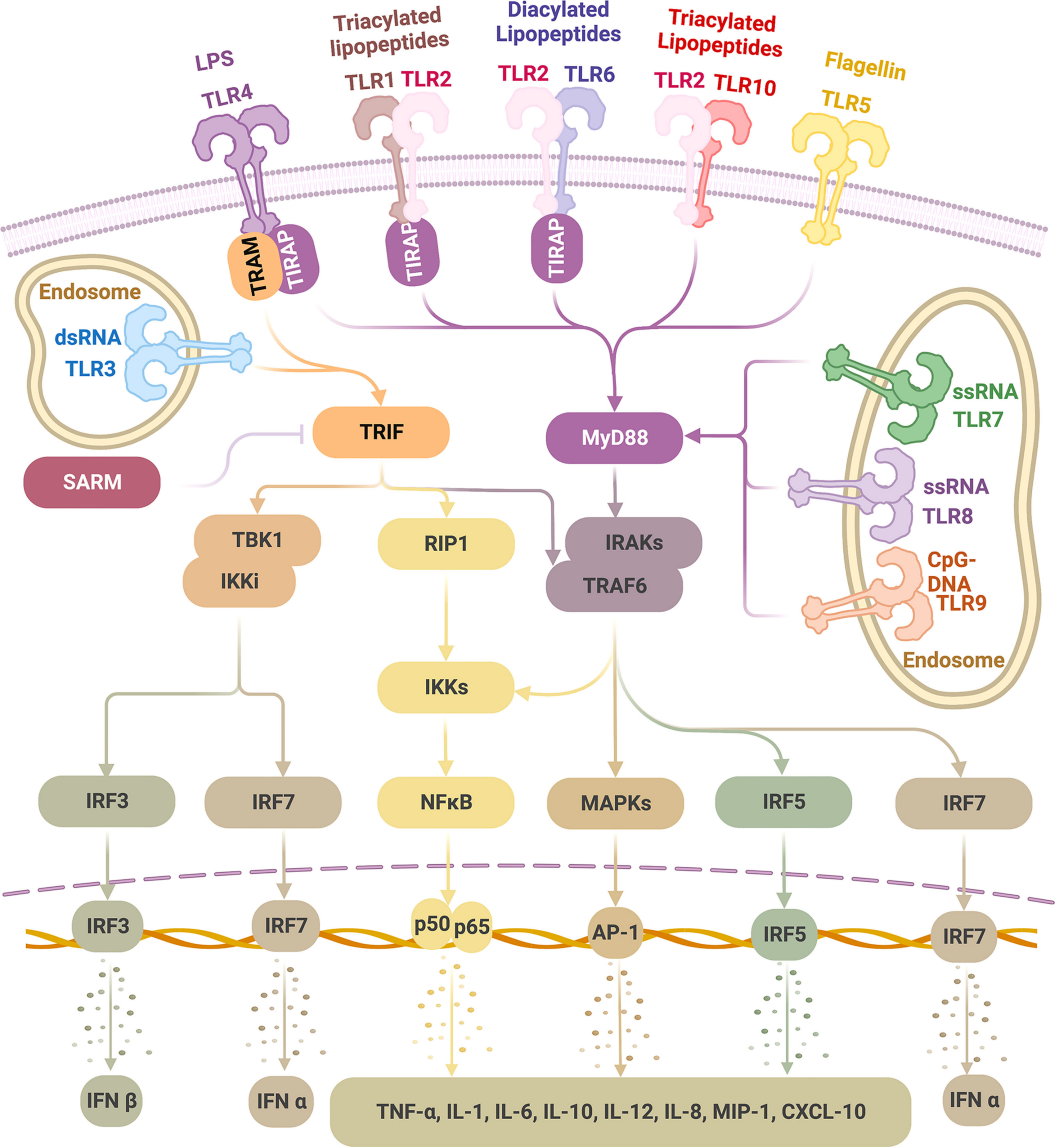
TLR ligands and signaling pathways Physiologically, TLRs are expressed on the cell membrane (TLR 1, 2, 4, 5, 6, 10) or the endosome (TLR3, 7, 8, 9). TLRs recognize a wide range of pathogen-associated molecular patterns (PAMPs). Upon ligation, dimerized TLRs can activate either MyD88 or TRIF pathways. TLR4 can activate MyD88 (with adaptor protein TIRAP) and TRIF (with adaptor protein TRAM) pathways; TLR3 activates TRIF alone, and TLR1/2, TLR2/6, TLR2/10, TLR5 as well as TLR7-9 activate MyD88 alone, all without the need of an adaptor protein. As a result, type I IFNs are induced *via* IRF3 and IRF7 activation and inflammatory cytokines *via* NF-kB, MAPKs, and IRF5 stimulation, which thus initiates a wide range of inflammatory and immune activities, giving the basis for therapeutic exploration on TLRs in tumor therapies. (This figure is created with BioRender.com).

In the ligand-binding, namely, activated status, TLRs tend to be dimerized (31). TLR2 alternatively forms heterodimers with TLR1, 6, or 10 (37, 38); each combination is associated with the specificity for recognizing a particular group of ligands, making TLR2 the most competent TLR in sensing a diverse range of PAMPs. Other TLRs tend to form homodimers (**Figure 1**) (39).

### LR ligands

TLRs do not necessarily distinguish between self and non-self, but they respond to “danger signals” in general; as long as the “signals” are encompassed within certain molecular patterns, both no matter whether exogenous or endogenous ligands (40).

TLR ligands (TLRLs) primarily fall into three categories (1): natural exogenous ligands (PAMPs) (2); natural endogenous ligands (DAMPs and secreted ligands) (3); synthesized agents.

Exogenous ligands, namely, PAMPs originate from broadly expressed components in bacteria, fungi, protozoa, parasites, and viruses (10, 41, 42); they are common and genetically conserved because of their significance to microbial survival and infectivity (1, 43). As sentinels of the host’s defense system, TLRs sense PAMPs as invading dangers and directly activate the innate immune system (44), meanwhile indirectly activating the adaptive immune system (43), thereby providing indispensable protection against pathogens. Studies on mice lacking each TLR have shown that each TLR has a different function in PAMP recognition and immune response (45). Cell surface TLRs mainly recognize microbial membrane components such as lipids, lipoproteins, and proteins. TLRs expressed in intracellular components, such as endosomes, recognize viral or bacterial nucleic acids (46, 47). Specifically, TLR1/2 recognizes triacylated lipopeptides from Gram negative bacteria (38, 39), TLR2/6 recognizes diacylated lipopeptide from mycoplasma (38, 48), and TLR2/10 recognizes microbial components shared by TLR1 (37). TLR3 ligands are double-stranded RNAs (dsRNAs) emerging from cytosol virus replication (13, 39). TLR4 binds lipopolysaccharide (LPS) from Gram negative bacteria (39, 49) and fungal mannanes (48).TLR5 ligates with bacterial flagellin (13).TLR7 and 8 ligands are both single-stranded RNAs (ssRNAs) from viruses (13, 39).TLR9 recognizes nonmethylated CpG motifs normally found in bacterial and viral DNA (13, 39).

On the other hand, endogenously derived DAMPs from cells and ECM also activate TLRs. DAMPs are generated from cellular components or fragments of extracellular macromolecules, which are typicallyinaccessible to TLR ligand-binding regions in homeostasis, yet exposed to TLRs in the case of cell necrosis (passive release), apoptosis (pulsative release) or extracellular matrix degradation (5, 16, 43, 50) following trauma, inflammation and tissue remodeling during development (43). Specifically, TLR2 recognizes HMGB1, β-defensin-3, serum amyloid A, and biglycan (51);TLR4 recognizes HMGB1, fibrinogen, saturated fatty acids, and hyaluronic acid fragments (51);TLR3 recognizes mRNA (52);TLR7 and TLR8 recognize ssRNA (53) and antiphospholipid antibody (54);TLR9 recognizes IgG-chromatin complexes (55).

In the tumor microenvironment (TME), DAMPs can be abundantly released by solid tumors (5, 56) and have a significant impact in the TME by adopting mechanisms of tissue repair [such as cell proliferation (57, 58), angiogenesis (56), and tissue remodeling (59)], contributing to tumor progression. Alternatively, just as PAMPs, DAMPs can also act as stimulators of immune responses, showing anticancer potential *in vitro* as well (56).

Synthetic TLRLs on the other hand have varied levels of similarity to natural ligands (41), designed for the purpose of disease treatment by manipulating different aspects of TLR functions. The synthetic TLRLs are in active pre-clinical and clinical investigation. The advances of TLRLs, including synthetic TLRLs, in therapeutic research are reviewed in the following section 5 and **Tables 1**–**3**.

**Table 1:** Recent pre-clinical therapeutic investigation incorporating TLR-targeting.

TLR	Ligand	Molecular nature	Treatment strategy	Condition	Therapeutic feature	Reference
Agonism						
TLR1/2	SMU-Z1	synthetic chemical compound	TLRa	leukemia	increased CD8+ T cells, NK cells and DCs	(60, 61)
	Pam3CSK4	triacylated lipopeptide	TLRa+ICB	melanoma	enhanced depletion of Tregs in tumor microenvironment	(62)
	Amplivant	triacylated lipopeptide	TLRa+vaccine +chemo/PDT	HPV16-related tumors	Better immune stimulatory effect compared to normal Pam3SCK4	(63)
	Diprovocim	synthetic chemical compound	TLRa+vaccine+ICB	melanoma	long-term antitumor memory, high frequencies of tumor-infiltrating leukocytes	(64)
TLR2	acGM-1.8	glucomannan polysaccharide	TLRa	sarcoma, melanoma	enhanced M1-polarization of macrophages; improved safety profile	(65)
	WCCP-N-b	galactan	TLRa	melanoma	enhanced M1-polarization of macrophages; reduced tumor cell viability	(66)
	HSV-1	inactivated virus	TLRa+ACT	acute myeloid leukemia	direct NK cell activation	(67)
TLR3	poly (I:C)	ds RNA mimic	TLRa	breast cancer	a 2-fold increase in the numbers of inflammatory cells expressing the myeloid markers	(68)
			TLRa	colon cancer, breast cancer	induction of cell death; increased CD8+ tumor infiltrating lymphocytes and CD8/Treg ratios	(69)
			TLRa+chemo	oral squamous cell carcinoma	down-regulated drug transporters P-gp and MRP-1; raised cytoplasmic concentration of cisplatin	(70)
			TLRa+chemo	lung cancer	impaired paclitaxel induced c-FLIP reduction; restored cancer cell apoptosis	(71)
			TLRa+CTT+ICB	multiple types of tumor	increased treatment sensitivity to ICB treatment	(72)
			TLRa+vaccine	leukemia, thymoma	induction of systemic cross-priming, systemic tumor-specific adaptive immunity, intratumoral CTL infiltration	(73)
			TLRa+vaccine +aCD40 Ab	colon cancer	increased ratio of tumor-specific T cell population; reduced side effects	(74)
			TLRa+vaccine +aCD40 Ab	prostate cancer	elevated antigen-specific cellular and humoral immunity	(75)
			TLRa (NP incorporated)	melanoma	elevated pro-inflammatory macrophage infiltration, decreased MDSCs, macrophage M1 polarization	(76)
			TLRa (NP incorporated)	melanoma	ROS generation, macrophage M1 polarization, elevated activated NK cells and T lymphocytes	(77)
			TLRa+vaccine (NP incoporated)	thymoma	lymph node targeted, higher uptake of drug in APCs, greater natural killer cell expansion/activation and CTL response	(78)
			TLRa+vaccine (NP incoporated)	HPV-induced malignancy	enhanced population of antigen-specific CD8+ T cells, reduced adverse effects	(79)
			TLRa+vaccine+GT (NP incorporated)	thymoma	incudtion of TADC maturation and activation, decreased STAT3 expression, abrogated immunosuppression, potent anti-tumor immune responses	(80)
	ARNAX	DNA-capped dsRNA	TLRa+vaccine	thymoma	enhanced infiltration of CD8+ T Cells and CD8a+ DCs, enhanced Th1-type anti-tumor immunity	(81)
TLR4	LPS	lipopolysaccharide	TLRa+RT+ACT	melanoma	augmentation of the antitumor activity of adoptively transferred CD8(+) T cells	(82)
	MPLA	monophosphoryl lipid A	TLRa+CTT	breast cancer	extensive upregulation of systemic and intratumoral APCs and NK cells	(83)
			TLRa+CLRa	breast cancer	stimulation of B-1 cells,rapid production of high levels of natural IgM reactive against tumor-associated antigens	(84)
			TLRa+proteasome inhibition	leukemia	enhanced tumor cell death	(85)
			TLRa+vaccine +NKTa	HPV-induced malignancy	elevated levels of lymphocyte proliferation, CTL activity, IFN- gamma, IL-4 and IL-12 responses	(86)
			TLRa+vaccine +CD4 Ta	breast cancer	enhanced tumor inhibition effect	(87)
			TLRa (NP incoporated)	melanoma	DC-targeting, induction of DC maturation and activation, promotion of anti-tumour T cell responses, and enhanced antigen cross-presentation	(88)
			TLRa+vaccine (NP incoporated)	melanoma	enhanced anti-tumor effects	(89)
			TLRa+vaccine (NP incoporated)	melanoma	promoted antigen retention in draining lymph nodes	(90)
	PELA	pentaerythritol lipid A	TLRa+vaccine (NP incoporated)	thymoma	enhanced stimulation of co-stimulatory molecules CD80/CD86 on DCs, significant expansion of the tumor-specific T cell population, enhanced Th1-biased immune response	(91)
	RGP	rehmannia glutinosa polysaccharide	TLRa+vaccine	melanoma colon cancer	increased IFN-γ secretion and CD8+ T cell response	(92)
	CIRP	protein	TLRa+vaccine ± ICB	melanoma, thymoma, colon cancer	CD8-dependent tumor rejection improved survival	(93)
	API5	protein	TLRa+DC vaccine	thymoma, cervical cancer, colon cancer	generation of antigen-specific CD8 + T cells and memory T cells	(94)
	HMGN1	protein	TLRa+vaccine	melanoma	tumor-specific, Th1-polarized immune responses	(95)
TLR5	flagellin	protein	TLRa+vaccine	genital cancer	local administration, induction of CD4+ and CD8+ cell recruitment as well as T cell activation-related gene expression in draining lymph nodes, systemic antigen-specific IFN-γ production	(96)
	flagellin	protein	TLRa+ACT	melanoma	cell therapy-bacterial flagellin-secreting DMF5(TLR5L) T cells: augmentation of T cell effector function and expansion, reduction of immunosuppressive cells	(97)
	Entolimod	protein	TLRa	colon cancer, breast cancer	stimulation of the NK-DC-CD8+ T-cell axis	(98)
TLR7	imiquimod	imidazoquinoline	TLRa	lymphoma	NK cell activation, induction and intra-tumoral infiltration of tumor-specific CD4(+) T cells	(99)
			TLRa+vaccine	HPV-induced malignancy	induction and recruitment of tumor-specific CD8+ T cells	(100)
			TLRa+chemo+ICB (NP incorporated)	breast cancer	induction of tumor-specific immune responses, enhanced DC maturation, elevated CD8(+) CTLs/Treg and CD4(+) Teff/Treg ratios	(101)
			TLRa+ICB+DCsti (NP incoporated)	menaloma	localized immunotherapy, safe antitumoral responses	(102)
			TLRa+PTD+ICB (NP incorporated)	colorectal cancer	generation of a pool of tumor-associated antigens, strong antitumor immunity and long-term immune memory	(103)
			TLRa+PTD+ICB (NP incorporated)	breast tumor	abscopal effects, tumors infiltrated by CD45(+) leukocytes	(104)
			TLRa+PTT+ICB (NP incorporated)	breast cancer, conlon cancer	enhanced DC activation, increased TILs, decreased Tregs, induction of immune memory, inhibition of metastasis	(105)
			TLRa+RT+ICB (NP incoporated)	colon cancer	relieved tumor hypoxia, effective inhibition of tumor metastases, long term immune memory	(106)
			TLRa+vaccine+ICB (NP incorporated)	melanoma	enhanced drug uptake by APCs, enhanced immune stimulation	(107)
	gardiquimod	imidazoquinoline	TLRa+ACT	liver cancer	direct activation of NK cells, enhanced maturation of DCs, promotion of NK-DC cross-talk	(108)
			TLRa+vaccine +aOX40/aCD40 Ab	melanoma	induction of anti-tumor CD4 T-cell responses, overcome immune tolerance to a self-tumor-associated antigen, enhancement of proliferative and antiapoptotic activities of CD4 T cells	(109)
	1V270	synthetic small molecule	TLRa	breast cancer, melanoma, lung cancer	activation of NK cells and tumor-specific CD8+ cell, inhibition of metastasis	(110)
			TLRa+ICB+IRE	pancreatic cancer	induction of abscopal effects	(111)
	SC1	synthetic small molecule	TLRa	murine tumor models	systemic induction of IFN I and activation of immune cells, increased leukocyte infiltration and activation, induction of tumor-specific CD8+ T cells	(112)
			TLRa	lymphoma	reversion of NK cell anergy and restoration of NK cell-mediated tumor cell killing	(113)
	SZU-101	synthetic small molecule	TLRa+chemo	lymphoma	strong cytokine production and enhanced cytotoxic T lymphocyte response	(114)
	let-7b (miRNA mimic)	RNA	TLRa (NP incorporated)	breast cancer	reversion of suppresive TME by reprogramming TAM and TIDC	(115)
	antigen-encoding RNAs	RNA	TLRa+vaccine (NP incorporated)	melanoma, colon cancer, lung cancer	high efficiency of drug uptake and encoded antigen expression by DC and macrophages, activating both innate and tumor-specific adaptive immunity	(116)
TLR7/8	resiquimod	imidazoquinoline	TLRa+ICB	colon cancer	generation of immune memory, induction of anti-tumor intratumoral myeloid cells	(117)
			TLRa+CD20 Ab	lymphoma	activation of NK cells, CD4+ T cells, CD8+ T cells and generation of immune memory	(118)
			TLRa+surgery	breast cancer, lung cancer	administration at the tumor resection site, increased NK, DC, and T cell activation, induction of IFN I	(119)
			TLRa (NP incoporated)	oral cancer, mast cell tumor	prolonged drug release and limited systemic immune toxity	(120)
			TLRa+ICB (NP incorporated)	colon cancer, melanoma	increased the proportion of TILs and sensitization of tumors to ICB	(121)
			TLRa+ICB (NP incorporated)	colon cancer, melanoma	incution of M1 polarization of TAMs, generation of immune memory and reversion of anti-PD-1 resistance	(122)
	resiquimod+ imiquimod	imidazoquinoline	TLRa+vaccine+ICB (NP incorporated)	melanoma, cervical cancer	M1 polarization of TAMs, stimulation of NK cells, increased TILs, and generation of immune memory	(123)
	522	imidazoquinoline derivative	TLRa+vaccine (NP incorporated)	melanoma	strong induction of antigen-specific CD8 T cell and NK cell responses	(124)
	522/528	imidazoquinoline derivative	TLRa+vaccine (NP incorporated)	melanoma, bladder and renal cell cancer	enhanced activation and expansion of DC and antigen-specific CD8 T cells	(125)
	modified mRNA	RNA	TLRa+vaccine (NP incorporated)	melanoma	potent stimulation of IFN-β and IL-12 from DCs and enhanced antigen presentation	(126)
TLR9	CpG ODN	DNA	TLRa	pancreatic cancer	enhanced macrophages antitumor activity by eliciting changes in the carbon metabolism	(127)
			TLRa	liver cancer	induction of iMATE in liver	(128)
			TLRa	brain metastatic melanoma	enhanced microglia-tumor contact and promotion of phagocytosis and killing	(129)
			TLRa+aOX40 Ab	lymphoma, colon cancer, breast cancer, melanoma	local and systemic anti-tumor T cell response	(130)
			TLRa+BTKi	lymphoma	induction of local and systemic T-cell dependent tumor eradication, generation of immune memory	(131)
			TLRa+chemo	glioma melanoma	increased tumor infiltration of macrophages and B cells, increased CTLs and generation of immune memory	(132)
			TLRa+ICB	lung cancer	formation of tertiary lymphoid structures adjacent to the tumors, infiltration of CTLs, DC expansion, activation of CTL supporting Th cells	(133)
			TLRa+ICB	breast cancer, colon cancer	reversion of PD-1 blockade resistance, enhanced CTL expansion and infiltration	(134)
			TLRa+ICB +aOX40 Ab	lymphoma	depletion of tumor-infiltrating Tregs and induction of local and systemic anti-tumor responses	(135)
			TLRa+iTreg	colon cancer	reduced infiltration of Tregs and increased CTL	(136)
			TLRa+RFA	thymoma	CpG enhanced RFA-induced CTL responses	(137)
			TLRa+RFA	liver cancer	enhanced antitumor T cell responses and Th1 cytokine production	(138)
			TLRa+STAT3i	leukemia	reduced arginase expression, restored T cell responses, elimination of leukemia stem/progenitor cells	(139)
			TLRa+STAT3i	lymphoma	generation of tumor-specific CD8/CD4 T cell immunity and immune memory	(140)
			TLRa+vaccine	renal cancer	rapid induction of tumor-specific CD8(+) T cells, induction of memory lymphocyte infiltration, and Th1-type immune response	(141)
			TLRa+vaccine	thymoma	neutropbil recruitment, activation of cDCs, T-cell priming in draining lymph nodes, increased CTL infiltration	(142)
			TLRa+vaccine	breast cancer	enhanced antigen-specific CD8+ T cell cytotoxicity	(143)
			TLRa+vaccine	melanoma	intracellular drug delivery, enhanced T cell response	(144)
			TLRa+vaccine +chemo	HPV-induced malignancies	reduced infiltration of MDSC and TAMs, increased antigen-specific CTL	(145)
			TLRa+vaccine+ICB	ovarian cancer	enhanced activation and expansion of antigen-specific CTLs, induction of memory precursor T cells, inhibition of Tregs and MDSCs	(146)
			TLRa+vaccine +NKTa	melanoma	optimized expansion of NKs and antigen-specific CTLs, increase of serum IL-12	(147)
			TLRa+vaccine (NP incorporated)	melanoma	TAM-targeted, M1 polarization of macrophages, increased T cell intra-tumoral infiltration and activation	(148)
			TLRa+vaccine (NP incorporated)	thymoma	potent CTL response	(149)
			TLRa+vaccine (NP incorporated)	thymoma	efficient delivery to DCs, induction of antigen-specific immune responses	(150)
			TLRa+vaccine (NP incorporated)	lymphoma	professional APC-targeted, induction of long-lasting, cytotoxic, antigen-Specific T cell responses	(147)
			TLRa+PTT (NP incorporated)	breast cancer	good safety profile, superior effectiveness towards the suppression of both primary and metastatic tumor over either single therapy alone.	(151)
			TLRa (NP incorporated)	melanoma	increased expression of proinflammatory factors and co-stimulatory factors	(152)
			TLRa+vaccine (NP incorporated)	melanoma	induction of ADCC, CTL responses	(153)
			TLRa+vaccine (NP incorporated)	melanoma	increased ROS generation, enhanced CTL proliferation	(154)
			TLRa+vaccine (NP incorporated)	melanoma	lymph node-targeted, effective drug delivery to endosomal TLR9 in APCs, enhanced cellular and humoral immune responses	(155)
			TLRa ± ICB (NP incorporated)	colon cancer	activation of intra-tumoral CD8 T cells, Th1-related gene induction	(156)
			TLRa+vaccine (NP incorporated)	melanoma	enhanced antibody response, Th1 polarization of Th responses	(157)
			TLRa+PDT (NP incorporated)	breast cancer	induction of continuous secretion of proinflammatory cytokines, maturation of DCs, activation and infiltration of T lymphocytes	(158)
			TLRa+vaccine (NP incorporated)		efficient intake by APCs, tumor-specific immune stimulation, minimal toxicity	(159)
			TLRa+chemo (NP incorporated)	glioblastoma	significantly enhanced tumor regression, prolonged survival, and generated immunological memory.	(160)
			TLRa+vaccine (NP incorporated)	prostate cancer	increased co-stimulatory molecule expression on DCs	(161)
			TLRa+vaccine (NP incorporated)	melanoma	lymph node-targeted, efficient DC delivery, induction of CTL expansion	(162)
			TLRa+vaccine (NP incorporated)	melanoma	enhanced tumor-specific Th1 and CTL responses, decreased splenic MDSCs and their intra-tumoral infiltration	(163)
	EnanDIM^®^	DNA	TLRa	colon cancer, melanoma, lymphoma, breast cancer	increased intra-tumoral T cell infiltration, generation of immune memory	(164)
	X-DNA	DNA	TLRa+chemo	colon cancer	enhanced activation of Th1 cells and DCs	(165)
	dSLIM	DNA	TLRa+vaccine	renal cancer	tumor-specific cellular and humoral immune responses	(166)
Multi-TLRs						
TLR2, 3	Pam3CSK4, poly (I:C)	triacylated lipopeptide, dsRNA mimic	TLRa+vaccine +aCD40 Ab (NP incorporated)	melanoma	efficient and selective delivery to DCs, improved priming of antigen-specific CD8+T cells	(167)
TLR2, 3, 7/8	LTA, poly (I:C), resiquimod	lipoteichoic acid, dsRNA mimic, imidazoquinoline	TLRa+aCS	pheochromocytoma	generation of immune memory and systemic anti-tumor immunity	(168)
TLR3,7	poly (I:C), imiquimod	dsRNA mimic, imidazoquinoline	TLRa	pancreatic cancer, head and neck squamous cancer, lung cancer	increased cytotoxicity and granzyme A/B production in γδ T cells	(169)
			TLRa+vaccine (NP incorporated)	melanoma	enhanced macrophage and DC functions, enhanced humoral and cellular immune responses, generation of immune memory	(170)
TLR3, 9	Poly (I:C), CpG ODN	dsRNA mimic, DNA	TLRa+chemo	melanoma	reduced arginase and IL-10 secretion from macrophages, good safety profile, enhanced recruitment and cytotoxicity of tumor-infiltrating NK cells	(171)
			TLRa+CTT/anti-MDSC Ab	melanoma	reduction of immunsuppressive molecule expression, increase in proinflammatory cytokine expression, increased NK cell recruitment and activation, good safety profile	(172)
			TLRa+vaccine (NP incorporated)	melanoma	effective therapeutic and prophylactic protection	(173)
TLR3, 8, 9	CU-CPT17e	synthetic small molecule	TLRa	cervical cancer, breast cancer	strong cytokine production and immune activation, effective inhibition of tumor cell proliferation	(174)
TLR4, 5	engineered bacteria	bacteria components	TLRa	colon cancer	engineered flagellin-secreting bacteria, TLR4- and TLR5-mediated immune activation, infiltration of abundant immune cells, M1 polarization	(175)
TLR4, 7	MPLA, imiquimod	monophosphoryl lipid A, imidazoquinoline	TLRa+vaccine (NP incorporated)	thymoma	improved DC cross-presentation, Th1-biased cytokine production, strengthened lymphocytes priming, generation of immune memory	(176)
			TLRa+vaccine ± ICB (NP incorporated)	thymoma	effective delivery to and activation of DCs, increased antigen-specific CD8+ T cells and memory T cells	(177)
TLR4, 9	MPLA, CpG ODN	monophosphoryl lipid A, DNA	TLRa+vaccine (NP incorporated)	melanoma	prolonged drug reactivation time, potent DC activation, effective T cell and macrophage activation	(178)
		monophosphoryl lipid A, DNA	TLRa+vaccine (NP incorporated)	melanoma	improved T, NKT, and NK cell infiltration, improved systemic immune stimulation	(179)
		monophosphoryl lipid A, DNA	TLRa+vaccine (NP incorporated)	melanoma cervical cancer	enhanced DC activation and humoral responses, improved antigen-specific CD8+ T cell responses	(180)
TLR7, 9	imiquimod, CpG ODN	imidazoquinoline, DNA	TLRa+vaccine	HPV-induced malignancy	intra-cheek immunization resulting in higher mobilization of mucosal CD8+ specific effector T cells in TdLNs and TME	(181)
	1V270, SD-101	synthetic small molecule small molecule, DNA	TRLa+ICB	head and neck cancer	induction of systemic adaptive immunity, increasde M1, tumor-specific CTL infiltration	(182)
**Antagonism**						
TLR2	OPN-301	protein	TLRant	gastric cancer	impeded initiation and growth of gastric cancer, significant suppression of CXCL2 and TNF-α genes	(183)
TLR7, 9	IRS-954	DNA	TLRant	liver cancer	significant prohibition of tumor growth	(184)
			TLRant	cholangiocarcinoma	inhibition of cancer cell proliferation *in vitro* and tumor growth *in vivo*	(185)
	HJ901	DNA	TLRant	diffuse large B cell lymphoma	significantly reduced TLR7- and TLR9-mediated cell proliferation in cell lines carrying a certain MyD88 mutation, prevention of tumor growth in mouse models	(186)
	chloroquine	chloroquine	TLRant	liver cancer	significantly impeded development of tumor	(184)
			TLRant	cholangiocarcinoma	inhibition of cancer cell proliferation *in vitro* and tumor growth *in vivo*	(185)

aCD40 Ab, agonistic anti-CD40 anti-body; aCS, activation of complement system; ACT, adoptive cell therapy; ADCC, antibody-dependent cellular cytotoxicity; anti-MDSC Ab, anti-MDSC antibody; aOX40 Ab, agonistic anti-OX40 anti-body; APCs, antigen presenting cells; API5, apoptosis inhibitor 5; BTKi, Bruton’s tyrosine kinase inhibition; CD20 Ab, anti-CD20 Ab; CD4 Ta, CD4+ T cell activation; cDC, conventional dendritic cell; chemo, chemotherapy; CIRP, cold-inducible RNA binding protein; CLRa, C-type lectin receptor (CLR) agonism; CTL, cytotoxic T lymphocyte; CTT, cytokine targeted therapy; DCs, dendritic cells; DCsti, DC cell stimulation; dSLIM, double stem loop immunomodulator with nonmethylated CG motifs; EnanDIM^®^, Enantiomeric DNA-based ImmunoModulator with nonmethylated CG motifs; ICB, immunocheckpoint blockade; IFN, inteferon; iMATE, intrahepatic myeloid aggregation for T cell expansion; IRE, irreversible electroporation; iTreg, inhibition of regulatory T cells; LTA, lipoteichoic acid; MDSC, myeloid derived suppressive cell; NK cells, natural killer cells; NKTa, natural killer T cell activation; NP incorporated, nanoparticle incorporated in drug delivery; ODN, oligodeoxynucleotide; PDT, photodynamic therapy; PTT, photothermal therapy; RFA, radiofrequency ablation; RT, radiotherapy; STAT3i, STAT3 inhibition; TADCs, tumor-associated DCs; TAM, tumor-associated macrophage; TdLNs, tumor draining lymph nodes; TIDC, tumor-infiltrated dendritic cell; TIL, tumor infiltrated lymphocytes; TILs, tumor-infiltrated lymphocytes; TLRa, TLR agonism; TLRant, TLR antagonism; TME, tumor microenvironment; vaccine, tumor antigen; vaccine, tumor antigen only vaccine. Tumor suppression has been observed in all of the listed studies, therefore not written in therapeutic features.

**Table 2:** Completed clinical trials applying TLR agonists.

TLR	Drug name	Other name	Phase	Indication	Therapeutic strategy	Route	Results	Trial number	Reference
TLR2	Hespecta (modified Amplivant)		I	HPV16-positive tumors or premalignant lesions	single use	intradermal	safe, induction of robust specific T-cell immunity	NCT02821494	(187)
TLR3	Poly ICLC	Hiltonol	II	melanoma, SCCHN, sacrcoma, non-melanoma skin cancers	single use	intratumoral/ intramuscular	well tolerated, systemic immune responses, achieving clinical benefit	NCT02423863	(188)
			II	solid tumors	+ vaccine	intramuscular		NCT02873819	
			I/II	Melanoma	+ vaccine + IFA	subcutaneous	activating humoral and T cell immunity	NCT01079741	
			I/II	Recurrent Glioblastoma	+ vaccine + Bevacizumab	intramuscular		NCT02078648	
			I/II	solid tumors	+ Durvalumab± Tremelimumab	intratumoral/ intramuscular		NCT02643303	
			I	AML, myelodysplastic syndrome	+ vaccine + chemotherapy	subcutaneous	induction of tumor antigen expression and cytotoxic antigen-specific T cells	NCT01834248	(187)
			I	AML	+ vaccine + Nivolumab+ chemotherapy	subcutaneous		NCT03358719	
			I	lung cancer	+ vaccine + Pembrolizumab + chemotherapy	subcutaneous	a good safety profile, induction of immune responses in nonsquamous NSCLC, needing future patient enrichment	NCT03380871	(189)
			I	melanoma	+ vaccine + IFA	intradermal/ subcutaneous	safe and effective, IFA enhanced T cell responses to peptide vaccines when added to TLR agonists.	NCT01585350	(190)
			I	pancreatic adenocarcinoma	+ vaccine	intratumoral		NCT01677962	
			I	solid tumors	+ vaccine	n.a.	good safety profile and immunogenicity	NCT02721043	(187)
			I	solid tumors	+ vaccine + Nivolumab	subcutaneous	good safety profile and immunogenicity	NCT02897765	(191)
			I	solid tumors	+ vaccines + Tadalafil	intramuscular	well-tolerated, reducing PDL1+macrophages, increasing activated tumor infiltrating T cells	NCT02544880	(192)
	Rintatolimod	Ampligen, Atvogen	I/II	recurrent ovarian, fallopian tube or primary peritoneal cancer	+ vaccines + IFA	intravenous		NCT01312389	
			II	colorectal carcinoma with metastatic disease to the liver	+ chemokine modulation	Intravenous		NCT03403634	
TLR4	LPS		I	melanoma	+ vaccine	intradermal/ subcutaneous	safe and effective vaccine adjuvant, IFA enhanced T cell responses to peptide vaccines when added to TLR agonists	NCT01585350	(190)
	GLA-SE	G100	I	melanoma	+ vaccine	intramuscular	well-tolerated, good immunogenecity	NCT02320305	
			I	Merkel Cell Carcinoma	+ surgery + RT	intratumoral	good safety profile, promising clinical efficacy	NCT02035657	(193)
			I	sarcoma	+ RT	intratumoral	effective local control of sarcoma, induction of local and systemic CD4+ T cell response	NCT02180698	(194)
	IDC-G305		II	melanoma, ovarian, renal cell or non-small cell lung cancer	single use	intramuscular	good safety profile and can generate antigen-specific immunity	NCT02015416	(195)
	GSK1795091	CRX-601	I	solid tumors	± pembrolizumab ± GSK3174998 ± GSK3359609	intravenous		NCT03447314	
	TriMix DC		I/II	melanoma	+ vaccine	Intranodal	Safe, but limited immunological and clinical response	NCT01530698	(196)
TLR5	entolimod	CBLB502	I	solid tumors	single use	intramuscular/subcutaneous		NCT01527136	
	Mobilan	M-VM3	I	prostate cancer	single use	intraprostate		NCT02654938	
TLR7	Imiquimod	R837, Aldara	III	vulvar Paget disease	+ paracetamol + Iidocaine	topical	82, 6% response rate; painkillers needed in 80% patients for side effects	NCT02385188	(197)
			III	VIN	single use	topical	a safe, effective alternative to surgery for HSIL patients; recommended as first-line treatment	NCT01861535	(198)
			II/III	CIN 2/3	single use (compare conization)	topical	inferior to conization in HPV clearance	NCT02130323	(199)
			II	CIN2/3	+ surgery	topical	promoting regression of cervical HSIL	NCT03233412	(200)
			II	breast carcinoma	single use	topical	effective treatment for breast carcinoma metastatic to skin/chest wall; well tolerated, promoting a pro-immunogenic tumor microenvironment	NCT00899574	(201)
			II	VIN2/3 and anogenital warts	single use	topical		NCT00941811	
			II	VIN2/3 and vulvar HSIL	+ vaccine	Topical		NCT03180684	
						
			I/II	breast carcinoma	+ radiation± chemotherapy	topical		NCT01421017	
			I	gastric cancer, breast cancer	+ vaccine+ chemotherapy + Sargramostim	topical		NCT02276300	
			I	Glioma (grade II)	± surgery ± chemotherapy ± RT + vaccine (tumor lysate)	topical		NCT01678352	
			I	glioma	+ vaccine	topical	grade 1 adverse effects; immune responses observed in 93.3% patients; enhanced IL-17 production and increased IDH1-specific T cells	NCT02454634	(202, 203)
			II	prostate carcinoma	+ vaccine + IFA	topical	safe and immunogenic; high number of administrations induced stronger immune response	NCT02293707	(204)
			I	prostate carcinoma	+ vaccines (two) + chemotherapy	topical		NCT02234921	
	TMX-101		II	bladder cancer	single use	intravesicle	mild adverse effects; significant urinary cytokine (IL-6, IL-18, IL-1β, IL-1ra, VEGF) increase with complete responders observed	NCT01731652	(205)
TLR7/8	852A		II	breast carcinoma; ovarian cancer; endometrial cancer; cervical cancer	single use	n.a		NCT00319748	
	Resiquimod	R848; S28463	I	melanoma	+ vaccine+ IFA	topical	safe, inducing both humoral and CD4+ T cell responses, insufficient to induce consistent NY-ESO-1-specific CD8+ T-cell responses	NCT00821652	(206)
TLR8	Motolimod	VTX-378, VTX-2337	II	epithelial ovarian cancer, fallopian tube cancer or primary peritoneal cancer	+ chemotherapy	subcutaneous	no improvement of clinical outcomes with adding Motolimod compared to placebo, subpopulation with ISR had longer OS compared to those without ISR in motolimod treated group	NCT01666444	(207)
			II	SCCHN	+ chemotherapy + Cetuximab	intravenous	no improvement in PFS or OS, whereas significant benefit in HPV-positive patients and those with ISR	NCT01836029	(208)
			I/II	ovarian cancer	+ Durvalumab + chemotherapy	subcutaneous		NCT02431559	
			I	squamous cell carcinoma	± anti-PD-1 (Nivolumab)	subcutaneous/ intratumoral		NCT03906526	
			I	SCCHN	+ Cetuximab	subcutaneous	enhanced NK cell responsiveness by adding VTX-2337	NCT01334177	(209)
			I	ovarian tumors	+ chemotherapy	subcutaneous	no dose-limiting toxicities, 2 subjects (15%) with complete responses, 7 subjects (53%) with disease stabilization	NCT01294293	(210)
TLR9	SD-101		I/II	lymphoma	+ Ipilimumab + RT	intratumoral		NCT02254772	
			I/II	solid tumors; lymphoma	+ Epacadostat + RT	intratumoral		NCT03322384	
	CMP-001	Vidutolimod	I	melanoma	+ Pembrolizumab	subcutaneous/intratumoral		NCT03084640	
			I	NSCLC	+ Atezolizumab+ RT	subcutaneous		NCT03438318	
	CpG7909	PF-03152676	I/II	Mantle cell lymphoma	+ vaccine+ autologous ACT + Rituximab + chemotherapy + Filgrastim	subcutaneous	safe, inducing antitumor CD8 T cell immune responses in 40% of patients, which were associated with favorable clinical outcomes	NCT00490529	(211)
	CpG7910		I/II	recurrent lymphomas	+ local RT	intratumoral	inducing systemic tumor-reactive memory CD8^+^ T cells	NCT00185965	(212)
	Tilsotolimod	IMO-2125	I	refractory solid tumors	single use	intratumoral	well tolerated, inducing immune checkpoint upregulation, activation of dendritic cells, and induction of Type 1 IFN signaling.	NCT03052205	(213)
	Lefitolimod	MGN1703	II	small-cell lung cancer	+ chemotherapy (platinum-based)	subcutaneous	well tolerated, no significance difference induced in main efficacy end point OS by lefitolimod	NCT02200081	(214)
	EMD 1201081		II	SCCHN	+ Cetuximab	subcutaneous	lack of clinical efficacy	NCT01040832	(215)
	DV281		I	NSCLC	+ Nivolumab	inhalation	well tolerated	NCT03326752	(216)
Multi-TLRs		
TLR2, 4	OM-174		I	solid tumors	single use	intravenous	well tolerated, 3/17 patients had 4 month disease stablization	NCT01800812	(217)
TLR2, 4, 9	BCG	Bacille Calmette-Guérin, Mycobacterium bovis	II	bladder carcinoma	+ Lenalidomide	intravesical		NCT01373294	
			II	bladder carcinoma	+ vaccine	intravesical		NCT02015104	
			II	lower urinary tract urothelial carcinoma	+ Sunitinib	intravesical	combination therapy associated with less recurrence and progression, no serious adverse effects	NCT00794950	(218)
			I/II	bladder carcinoma	+ Atezolizumab	intravesical		NCT02792192	
			I	bladder carcinoma	+ Rapamycin	intravesical	well-tolerated, combination thereapy enhancing BCG-specific γδ T cell immunity and increasing urinary cytokines	NCT02753309	(219)
TLR3, TLR7/8	poly-ICIC; resiquimod	Hiltonol; R848, S28463	I/II	advanced tumor refractory to conventional treatment	+ vaccine	epidermal topical/ subcutaneous	safe, inducing both NY-ESO-1-specific humoral and cellular immunity in NY-ESO-1 expressing patients, disease stabilization or tumor regression observed	NCT00948961	(220)
TLR3, TLR7	poly-ICLC; imiquimod	Hiltonol; R837, Aldara	I	glioblastoma	+ autologous glioma lysate-pulsed DC	intradermal	safe, mesenchymal gene expression profile associated with responsiveness to immunotherapies	NCT00068510	(221)
TLR4, 9	AS-15	MPL, QS-21, CpG ODN	II	metastatic melanoma	+ vaccine + high-dose IL-2	intramuscular	25% response rate; similar toxicity to high-dose IL-2 therapy alone; increased infiltrating T cells in the untreated tumor correlates with patient responsiveness	NCT01266603	(222)

ACT, adoptive cell therapy; ALL, acute lymphoblastic leukemia; AML, acute myeloid leukemia; Autologous hematopoietic stem cell transplant (HSCT); CIN, Cervical intraepithelial neoplasia; CLL , Chronic lymphocytic lymphoma; DCIs, donor lymphocyte infusions; HSIL, High Grade Squamous Intraepithelial Lesion; ICB, immunocheckpoint blockade; IFA, incomplete freund’s adjuvant; ISR, injection site reactions; NSCLC, non-small cell lung cancer; RFA, Radiofrequency ablation; RT, radiotherapy; SCCHN, squamous cell cancer of the head and neck; VIN, Vulvar Intraepithelial Neoplasias.

**Table 3:** Active and currently recruiting clinical trials incorporating TLR agonism.

TLR	Drug name	Other name	Phase	Indication	Therapeutic strategy	Route	Status	Trial number	Reference
TLR2	XS15		I	CLL	± multi-peptide vaccine + Ibrutinib	n.a.	recruiting	NCT04688385	
	1928T2z CAR-T cells	WZTL002-1	I	B-cell lymphoma	single use	intravenous	recruiting	NCT04049513	
TLR3	Poly ICLC	Hiltonol	II	brain tumors	+ vaccine	n.a	active, not recruiting	NCT01204684	
			I/II	melanoma	+ vaccine+Tetanus peptide ± IFA	intradermal/ subcutaneous	active, not recruiting	NCT02126579	
			I/II	recurrent ovarian, fallopian tube, or primary peritoneal cancer	+ vaccine+ GuaDecitabine + Atezolizumab	subcutaneous	active, not recruiting	NCT03206047	
			I/II	B-cell Lymphoma	+ rhuFlt3L/CDX-301 + RT	intratumoral	recruiting	NCT01976585	
			I/II	melanoma	+ vaccine +aCD40 Ab	subcutaneous/ intradermal	recruiting	NCT04364230	
			I/II	metastatic colon cancer	+ Pembrolizumab	intramuscular	recruiting	NCT02834052	
			I	multiple myeloma	+ vaccine+ Citarinostat ± Lenalidomide	n.a.	recruiting	NCT02886065	
			I	breast carcinoma	+ vaccine + Durvalumab	intramuscular	active, not recruiting	NCT02826434	
			I	breast carcinoma	+ vaccine + Pembrolizumab	n.a.	active, not recruiting	NCT03362060	
			I	glioma	+ a peptide vaccine ± aCD27 Ab	subcutaneous	active, not recruiting	NCT02924038	
			I	glioma	+ a peptide vaccine	n.a.	active, not recruiting	NCT02960230	
			I	glioma	+ vaccine + surgery	subcutaneous	active, not recruiting	NCT02549833	
			I	lung cancer	+ vaccine	subcutaneous	active, not recruiting	NCT03300817	
			I	prostate cancer	+ surgery	intramuscular	recruiting	NCT03262103	
	Rintatolimod	Ampligen, Atvogen	I/II	recurrent platinum-sensitive ovarian cancer	+ Pembrolizumab+ chemotherapy	intraperitoneal	recruiting	NCT03734692	
			I/II	cancer patients with mild or moderate COVID-19 infection	+ IFN α-2b		recruiting	NCT04379518	
	BO-112		II	melanoma	+ Pembrolizumab	intratumoral	active, not recruiting	NCT04570332	
TLR7	Imiquimod	R837, Aldara, UGN-201	III	anal intraepithelial neoplasia (HIV patients)	single use	topical	recruiting	NCT02059499	
			III	basal cell carcinoma	+ curettage	topical	active, not recruiting	NCT02242929	
			III	basal cell carcinoma at high risk (prevention study)	single use	topical	not yet recruiting	NCT05212246	
			II	basal cell carcinoma	+ sonidegib ± surgery	topical	recruiting	NCT03534947	
			II	CIN	single use/ + vaccine	topical	recruiting	NCT02864147	
			II	CLL	+ vaccine + Lenalidomide	topical	recruiting	NCT02802943	
			I	bladder cancer	+ surgery	intravesical	recruiting	NCT05055050	
			I	recurrent bladder cancer	+ zalifrelimab	Intravesical	recruiting	NCT05375903	
			I	CIN	+ chemotherapy	topical	active, not recruiting	NCT03196180	
			I	oral cancer	single use	topical	recruiting	NCT04883645	
			I	solid tumors	+ ultrasound ablation± Pembrolizumab/Atezolizumab	topical	recruiting	NCT04116320	
			I	solid tumors	+ vaccine	topical	recruiting	NCT03872947	
			I	squamous cell carcinoma	+ chemotherapy	topical	recruiting	NCT03370406	
			I	melanoma	+ vaccine + Toripalimab + GM-CSF	topical	recruiting	NCT04072900	
			I	melanoma	+ Pembrolizumab	topical	active, not recruiting	NCT03276832	
			I	recurrent glioblastoma	+ vaccine + hP1A8	topical	active, not recruiting	NCT04642937	(223)
			I	glioma (Newly Diagnosed H3-mutated Glioma)	+ vaccine + Atezolizumab	topical	not yet recruiting	NCT04808245	
	SHR2150		I/II	metastatic solid tumor	+ chemotherapy + anti-PD-1 ab + /anti-CD47 ab	oral	recruiting	NCT04588324	
	DSP-0509		I/II	neoplasms	± Pembrolizumab	intravenous	recruiting	NCT03416335	
	BNT411		I/II	solid tumors	± chemotherapy ± Atezolizumab	intravenous	recruiting	NCT04101357	
	RO7119929		I/II	HCC, biliary tract cancer, secondary liver cancer	± Tocilizumab	oral	active, not recruiting	NCT04338685	
	LHC165		I	solid Tumors	± PDR001	intratumoral	active, not recruiting	NCT03301896	
	RNA-lipoplexes	RNA-LPX	I	melanoma patients	single use	intravenous	active, not recruiting	NCT02410733	(116)
TLR7/8	Resiquimod	R848; S28463	II	brain tumors	+ vaccine	n.a	active, not recruiting	NCT01204684	
			I/II	melanoma	+ vaccines ± IFA	intradermal/subcutaneous	active, not recruiting	NCT02126579	
	TransCon	prodrug of resiquimod	I/II	solid tumors	± Pembrolizumab	intratumoral	recruiting	NCT04799054	
	BDC-1001		I/II	HER2 positive solid tumors	+ vaccine ± Pembrolizumab	n.a	recruiting	NCT04278144	
	BDB001		I	solid tumors	single ± Pembrolizumab	n.a	active, not recruiting	NCT03486301	
			I	solid tumors	+ Atezolizumab	n.a	active, not recruiting	NCT04196530	
	BDB018		I	advanced solid tumor	± Pembrolizumab	n.a	recruiting	NCT04840394	
TLR8	sbt-6050		I	HER2 positive solid tumors	± Pembrolizumab	n.a	active, not recruiting	NCT04460456	
TLR9	SD-101		II	prostate cancer	+ Pembrolizumab + radiation	intratumoral	recruiting	NCT03007732	
			I/II	lymphoma	+ Ibrutinib + RT	intratumoral	active, not recruiting	NCT02927964	
			I	lymphoma	+ BMS-986178 + RT	intratumoral	active, not recruiting	NCT03410901	
			I	pancreatic adenocarcinoma	+ Nivolumab + radiation	intratumoral	active, not recruiting	NCT04050085	
			I	solid malignancies	+ BMS 986178	intratumoral	active, not recruiting	NCT03831295	
			I	liver metastatic uveal melanoma	+ Nivolumab/Ipilimumab	pressure-enabled hepatic artery infusion/ intrahepatic	recruiting	NCT04935229	
	Tilsotolimod	IMO-2125	II	malignant melanoma	single use	intradermal	recruiting	NCT04126876	
			II	solid tumors	+ Ipilimumab + Nivolumab	intratumoral	active, not recruiting	NCT03865082	
			I	advanced solid malignancies	+ Ipilimumab + Nivolumab	intratumoral	active, not recruiting	NCT04270864	
	CMP-001	Vidutolimod, ARB-1598, CMP-001, CYT-003	III	multiple types of tumors	+ Avelumab + Utomilumab + PF04518600	subcutaneous/ intratumoral	recruiting	NCT05059522	
			II/III	melanoma	+ Nivolumab	intratumoral	active, not recruiting	NCT04695977	
			II	advanced cancer	+ Avelumab + Utomilumab + PF-04518600	n.a	active, not recruiting	NCT02554812	
			II	melanoma	+ Nivolumab	subcutaneous/ intratumoral	recruiting	NCT04401995	
			II	melanoma	+ surgery + Pembrolizumab	subcutaneous	recruiting	NCT04708418	
			II	squamous cell carcinoma of head and neck	+ Pembrolizumab	subcutaneous/ intratumoral	active, not recruiting	NCT04633278	
			II	melanoma	+ Nivolumab	intratumoral	active, not recruiting	NCT04698187	
			II	multiple tumor types	+ Cemiplimab-rwlc	subcutaneous/ intratumoral	recruiting	NCT04916002	
			II	Triple Negative Breast Cancer	+ RT	subcutaneous	recruiting	NCT04807192	
			II	melanoma, lymphnode cancer	+ Nivolumab	intratumoral	active, not recruiting	NCT03618641	
			I/II	advanced pancreatic cancer and other solid tumors	+ Ipilimumab	subcutaneous/ intratumoral	recruiting	NCT04387071	
			I/II	lymphoma	+ Pembrolizumab	intratumoral	recruiting	NCT03983668	
			I	colorectal neoplasms malignant, liver metastases	+ Nivolumab+ Ipilimumab ± RT	subcutaneous+ intratumoral	Active, not recruiting	NCT03507699	
			I	melanoma	+ Pembrolizumab	intratumoral	active, not recruiting	NCT02680184	
	DUK-CPG-001		II	myeloid malignancies, lymphoid malignancies	+ NK cell enriched DCIs	intravenous	recruiting	NCT02452697	
	Lefitolimod	MGN1703	I	advanced cancers, melanoma	+ Ipilimumab	subcutaneous	active, not recruiting	NCT02668770	
TLR2, 4, 9	BCG	Bacille Calmette-Guérin, Mycobacterium bovis	III	bladder carcinoma	single use	intravesical/ intradermal	active, not recruiting	NCT03091660	
			II	bladder carcinoma	+ Nivolumab ± BMS-986205	intravesical	active, not recruiting	NCT03519256	
			II	bladder carcinoma	+ ALT803, an IL-15 superagonist	intravesical	recruiting	NCT03022825	
			I/II	bladder carcinoma	+ ALT803	intravesical	recruiting	NCT02138734	
			I/II	bladder carcinoma	+ Durvalumab	intravesical	recruiting	NCT03317158	
			I	bladder carcinoma	+ Pembrolizumab	intravesical	active, not recruiting	NCT02808143	
			I	liver metastatic colorectal cancer	+ chemotherapy+RFA + GM-CSF	intralesion	not yet recruiting	NCT04062721	
TLR3, TLR7/8	poly-ICIC; resiquimod	Hiltonol; R848, S28463	I/II	melanoma	+ vaccine +Tetanus peptide ± IFA	intradermal/ subcutaneous	active, not recruiting	NCT02126579	

ACT, adoptive cell therapy; ALL, acute lymphoblastic leukemia; AML, acute myeloid leukemia; Autologous hematopoietic stem cell transplant (HSCT); CIN, Cervical intraepithelial neoplasia; CLL , Chronic lymphocytic lymphoma; DCIs, donor lymphocyte infusions; HSIL, High Grade Squamous Intraepithelial Lesion; ICB, immune checkpoint blockade; IFA, incomplete freund’s adjuvant; ISR, injection site reactions; NSCLC, non-small cell lung cancer; RFA, Radiofrequency ablation; RT, radiotherapy; SCCHN, squamous cell cancer of the head and neck; VIN, Vulvar Intraepithelial Neoplasia.

## TLR signaling

TLR signaling is initiated after TLR-ligand binding, which causes conformational changes, leading to the recruitment of four major adaptor proteins (**Figure 1**) (41). Each of these proteins contain a TIR domain that is homologous to the intracellular region of TLRs; all TLRs transmit signals through at least one of those proteins: myeloid differentiation factor 88 (MyD88), TIR domain-containing adaptor protein (TIRAP, also identified as MyD88 adaptor-like protein, MAL), TIR domain-containing adaptor protein-inducing interferon (IFN)-β (TRIF, also identified as TIR domain-containing adaptor molecule 1, TICAM1), and TRIF-related adaptor molecule (TRAM, also identified as TICAM2 and TIR-containing protein, TIRP) (1, 10, 41, 224, 225). A fifth TIR-domain exists, containing adaptor protein- sterile alpha and HEAT/armadillo motif protein (SARM), but contrary to the other four adaptors with functions of TLR signaling stimulation, it negatively regulates TLR signaling by inhibiting the downstream of TRIF pathway (225, 226).

TLR signaling can be divided into two pathways according to the TIR-containing adaptor used: the MyD88 pathway (MyD88-dependent pathway) and the TRIF pathway (MyD88-independent pathway) (227). TLR4 is the only TLR that activates both pathways; TLR3 relies only on the TRIF pathway; the remaining TLRs, TLR1-2, TLR5-10, activate solely the MyD88 pathway (10, 24, 37, 227). When initiating these pathways, some TLRs require an accompanying adaptor molecule. TLR4 and TLR2 (after dimerized with TLR1 or TLR6 but not TLR10) require TIRAP to recruit MyD88. TLR4 requires TRAM to bridge to TRIF (227–229). However, TLR5 and TLR7-9 can independently interact with MyD88 (227), and TLR3 depends solely on TRIF (228, 230).

In the MyD88 pathway, a series of downstream molecules are sequentially activated following recruitment of MyD88, including but not limited to interleukin-1 receptor-associated kinases (IRAKs), tumor necrosis factor (TNF) receptor-associated factor 6 (TRAF6), interferon (IFN) regulatory factor 5 (IRF5), receptor-interacting protein kinase 1 (RIP1), nuclear factor κB (NF-κB) inhibitor (IκB) kinase (IKK) complex, finally leading to the activation of mitogen-activated protein kinases (MAPKs) (ERK, JNK, p38) and NF-κB, which induces a wide range of pro-inflammatory cytokines (227, 228). In addition, in a cell-specific manner, following TLR7-9 ligation, the stimulated MyD88 pathway in plasmacytoid dendritic cells (pDCs) can activate IRF7, which subsequently leads to IFNα production (227).

In the TRIF pathway, IRF3 is activated and translocated into the nucleus, consequently resulting in IFNβ induction (227, 228). TLR3 and TLR4 can signal through the IRF3/IFNβ pathway (231, 232). In addition, TRIF can also stimulate RIP1 and TRAF6, leading to activation of MAPKs and NF-κB to induce pro-inflammatory cytokine production (227, 228).

Besides typeIIFN (IFNα, IFNβ) and inflammatory cytokines (TNFα, interleukin-1 (IL-1), IL-6, IL-10, IL-12, etc.), TLRs also induce many different chemokines (IL-8, macrophage inflammatory protein (MIP)-1, IP-10, etc.) and microRNAs (miRNAs) (10, 41, 233–235). In cells, TLR activation may lead to apoptotic, proliferative, or differentiative responses (41). In tissues, TLR signaling can result in inflammation, immune responses, and tissue repair (236).

## TLR expression

In humans, TLRs have a broad expression in various tissues, with the most diversity in locations involved in immune function, such as the spleen and peripheral blood, and locations in constant contact with microbes, such as the lung and the gastroenterological tract (237). Other locations expressing TLRs include the female reproductive tract, urinary tract, skin, neural system, and vascular system (238–240).

Specific to cell types, TLRs can typically be found in immune cells, such as DCs, monocytes, macrophages, granulocytes, NK cells, mast cells, and lymphocytes. Other non-immune cells also express TLRs, including endothelial cells, epithelial cells, fibroblasts, glial cells, astrocytes, keratinocytes, vascular smooth muscle cells, and sperm cells (3, 10, 33, 238, 239, 241, 242).

Recent evidence has shown that TLRs are expressed in diverse tumor cell populations (including tumor stem cells) together with cancer-associated fibroblasts, tumor-associated macrophages, myeloid-derived suppressor cells (MDSCs), regulatory T cells (Tregs), and adipocytes in the TME, participating in both promotion and inhibition in tumor growth (4, 10, 243).

During the process of malignant transformation, the level of TLR expression tend to elevate in transformed cells (including tumor cells) (24, 238, 244); meanwhile, the expression of TLR2, 4, 5, which is normally on cell membrane, increases in cytosol in a diffused manner (25, 245).

The expression patterns of TLRs and the downstream molecules are varied and regulated by multiple factors (237). Understanding these factors is vital for better exploitation in pre-clinical/translational research in TLR-targeted anti-tumor therapies.

TLR expression varies among distinctive species. For example, in humans, TLR9 is primarily expressed in B cells, whereas in mice, it can be found in B cells, monocytes, macrophages, and plasmacytoid DCs (pDCs) (246). Thus, cross-species interpretation of TLR research data should be carried out with caution.

Cell differentiation and activation status can also influence TLR expression. The differentiated human monocytic cell line THP-1 expresses increased TLR1, 4, 6, 7, 8, and MyD88 compared to undifferentiated THP-1 cells (33). E. coli-stimulated monocytes and granulocytes present different expression profiles of TLR1-10 (237). Phorbol myristate acetate/ionomycin-activated cytotoxic T cells (CTLs) showed increased TLR2 expression compared to unstimulated CTLs (247).

Ligands are another major factor affecting TLR expression. A specific TLR agonist may influence the expression of the TLR that it is ligating to and other TLRs as well. For example, the TLR4 agonist LPS elevated TLR1-8 expression in THP-1 cells, not just TLR4 (237). In addition, different ligands may differentially regulate the expression of a particular TLR. While TLR3 expression is upregulated by LPS, it is down-regulated by synthetic bacterial lipopeptide - a TLR2 agonist (237). Hence, cell localization related to potential ligand accessibility can impact TLR expression (39). As the case with TLR2 expression in B cells, only a restricted portion of peripheral blood B cells express TLR2 (248), but different developmental stages of tonsillar B cells all express TLR2 (249).

Cytokines are also engaged in the regulation of TLR expression. Many cytokines have been reported to increase TLRs expression level, including IL-6 and IFN-γ (237).

TLR activation can initiate negative feedback on TLR expression (237), and there is also internal balance among different expression of different TLRs (250, 251).

The factors discussed above indicate a complex network regulating TLR expression. This results in varied spectrums of TLR expression in respective cell groups and varied TLR-mediated responses, contributing to the heterogeneity of neoplasia, while also providing the basis for understanding the evidence for both anti-tumor and pro-tumor TLR-mediated activities. TLRs expressing in cancer cells could lead to tumor cell proliferation and induce cytokines that suppress immune activities; TLRs expressing on immune cells, however, could elicit Th1-based immunity, immune memory, and suppress tumor metastasis (252). The TLR expression in different tumor tissues is associated with patients’ outcome. Urban-Wojciuk et al. (252) have reviewed the association. In their review, the same TLR expressing in different types of cancers, could show opposite clinical correlations. TLR5, 7, 8, and 9 correlate with better clinical outcomes (high immune cell marker expression and/or longer survival), while TLR4, 7, and 9 are associated with poorer outcomes (advanced tumor stage, poor differenciation, and shorter survival) (252).

## Recent advances of TLR-targeted tumor therapies

TLR-targeted therapies exert anti-tumor effects mainly by exploiting the potential of TLRs in the enhancement of both innate and adaptive immunity, and the induction of apoptosis in TLR-expressing tumor cells (41).

**Figure 2:**
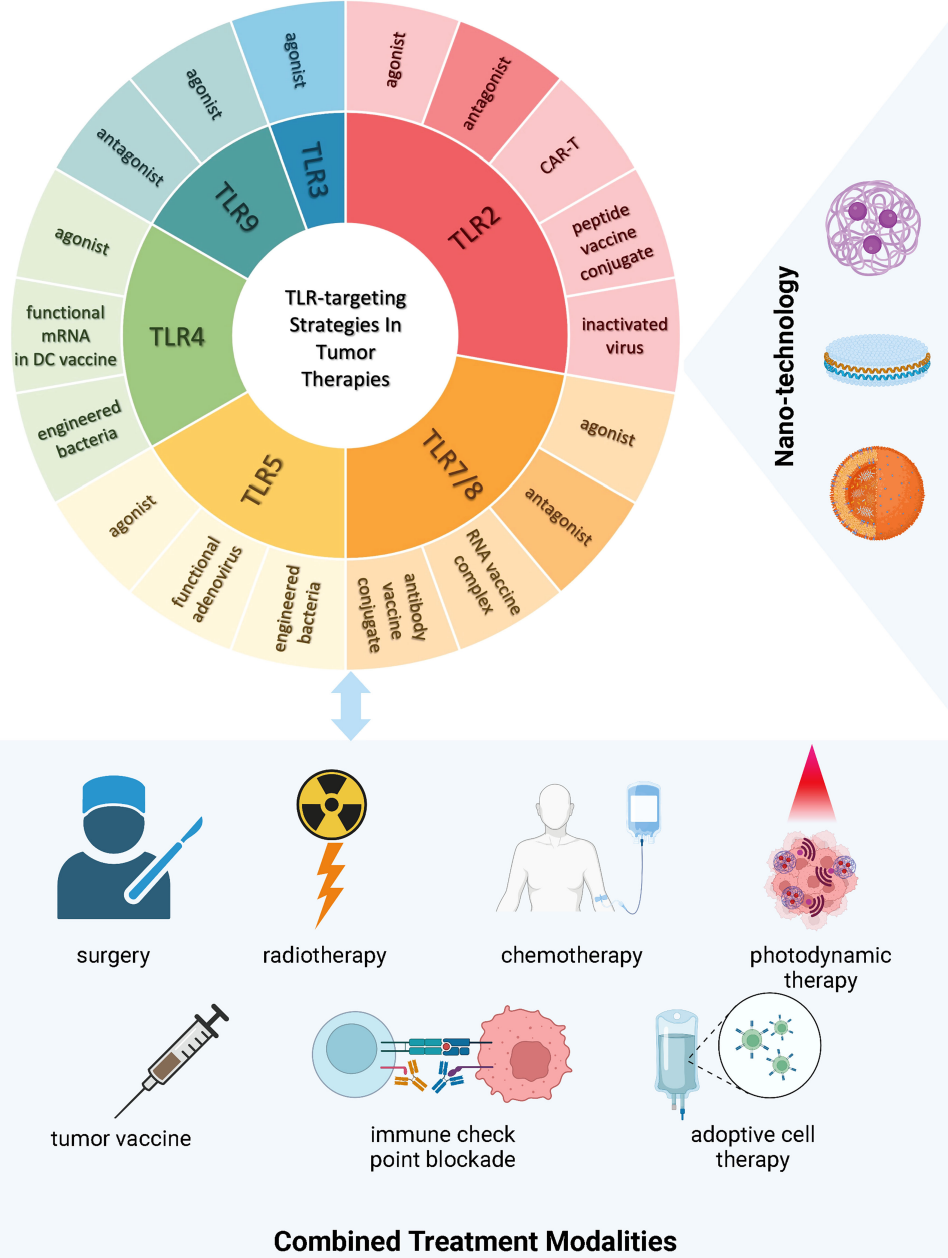
TLR-targeting Strategies and Combined Treatment Modalities in Tumor Therapies Current strategies targeting TLRs in tumor therapies encompass the development of novel TLR agonists, inactivated virus, engineered bacteria, functional RNAs expressing the TLR and/or its ligand, vaccine conjugates, modified T cells with active motifs of the TLR, and TLR antagonism. There is a trend of incorporating nanotechnology into TLR drug manufacturing and delivery to improve treatment efficacy and safety. Increasing evidence demonstrates the insufficiency of single intervention in circumventing immunosuppression incurred by tumor progression, hence leading to both pre-clinical and clinical research focusing on multi-agent and multi-modality treatment. (This figure is created with BioRender.com).

In this section, we introduce advances in pre-clinical (mainly from the past decade) as well as clinical research in tumor therapies targeting TLR. The TLR-targeting strategies and treatment modalities applied in both pre-clinical and clinical tumor therapeutic studies are summarized in **Figure 2**.

Pre-clinically, classic TLR agonists are being further explored in novel treatment strategies, and newly synthesized or naturally extracted TLR-stimulating agents are reported for improved immune activation ability and lowered toxicity; novel strategies of TLR agonism, aside from TLR agonists, have also been investigated (**Table 1**).

There have been many clinical trials completed involving TLRs, and some with promising results (**Table 2**). The ongoing clinical trials are continuously pushing the boundary of TLR-targeted therapy from bench to bedside (**Table 3**). Recent advances in clinical trials focusing on therapeutic interventions of tumors were summarized from critically reviewing *clinicaltrials.gov* and *ncbi.nlm.nih.gov/pubmed*.

### TLR 2

In tumors, TLR2 has been found to enhance T cell immunity (253, 254) and suppress the accumulation of immunosuppressive T regulatory cells (Tregs) (62) and MDSCs (255). TLR2 also induces the anti-tumor M1-like macrophage polarization (66). The immune stimulating potential of TLR2 has been utilized in both basic and clinical therapeutic research.

Recently, there has been pre-clinical data (**Table 1**) demonstrating tumor inhibition effects by different TLR2L, including triacylated lipopeptides, synthetic chemical compounds, glucomannan polysaccharide, naturally extracted compound, and inactivated virus. In those studies, TLR2Ls are either investigated alone or in combination with tumor vaccines, immune checkpoint inhibition (ICB), chemotherapy, photodynamic therapy(PDT), or adoptive cell transfusion (ACT). In general, enhanced immune activation and suppressed tumor cell viability were observed in these studies (**Table 1**).

The number of clinical trials with sole TLR2 agonism in tumor immunotherapy is limited. It is noteworthy that Amplivant, the modified Pam3CSK4, can conjugate synthetic long peptides (SLPs) and elicit stronger DC and T cell stimulation in preclinical models compared to Pam3CSK4 (63). In 2021 the first-in-human phase I clinical trial NCT02821494 showed HPV16 E6 SLP-conjugated Amplivant induced robust HPV16-specific T cell immunity with good safety profile (187).XS15 is a TLR1/2 agonist, applied as an adjuvant to a multi-peptide vaccine scheme is in currently active phase I trial NCT04688385 (256).

TLR2 agonism has been applied in chimeric antigen receptor T cell (CAR-T) technology. In trial NCT02822326, a TLR2 Toll/IL-1 domain is incorporated in CAR-T, and the results showed that the tumor-targeting CD19-CAR-T2 cells augmented anti-leukemia responses in relapsed or refractory B cell acute lymphoblastic leukemia (B-ALL) patients with extramedullary involvement, competent in the eradication of extramedullary leukemia cells (257). Another similar phase I trial NCT04049513 is currently investigating the efficacy of 1928T2z CAR-T cells in treating B cell lymphoma.

In general, the Amplivant-SLP compounds are promising for further research for their early clinical data in terms of safety and efficacy of immune stimulation. Also, they have a great potential in treating different tumors as SLPs can be highly variable.

Clinical trials with multi-TLR activation, including TLR2, are discussed in the below sections.

### TLR 3

TLR3 actively regulates multiple aspects of tumor development. Either directly or indirectly, TLR3 triggers both innate and adaptive immune responses (258, 259);, it suppresses tumor cell proliferation and promotes tumor cell apoptosis (260–262); it regulates tumor angiogenesis (263); it has also been found to enhance chemosensitivity (70) and radiosensitivity (264). By initiating these mechanisms, TLR3 agonists have shown promising therapeutic value.

In recent years, pre-clinical investigations of TLR3-targeted therapy (**Table 1**) have focused on combination therapy of TLR3 agonism with other treatment modalities, including chemotherapy, tumor vaccines, and ICB. There have also been studies examining nanoparticle incorporated TLR3 targeted therapies. Poly (I:C) has been used in most studies as the TLR3-targeting agent, while ARNAX, a new DNA-capped dsRNA, has also been applied. These treatment strategies aim to improve immune stimulation and reduce treatment side effects, thus achieving better therapeutic results.

Three TLR3 agonists, Poly ICLC (Hiltonol), Rintatolimod (Ampligen), and BO-112 have progressed for assessment in clinical trials (**Tables 2** and **3**) as cancer treatments.

Poly ICLC is carboxymethylcellulose, polyinosinic-polycytidylic acid, and poly-L-lysine double-stranded RNA, administratedintratumorally (i.t.), intramuscularly (i.m.), or subcutaneously (s.c.) in cancer treatments of different malignant stages, including primary, recurrent, and metastatic. The various types of cancer involved range from hematopoietic malignancies, skin cancers, sarcoma, squamous cell carcinomas, breast cancer, neurological malignancies, gastroenterological tumors, female reproductive tract malignancies, and urinary tumors (**Tables 2** and **3**). In these cancer treatments, Poly ICLC is mostly used in combination with traditional therapies (surgery, chemotherapy, or radiotherapy), immunotherapies (tumor vaccine, immune co-stimulation, or ICB), or other synthesized anti-tumor agents. Most of the trials are active or undergoing recruitment. One completed phase II trial (NCT02423863) aiming at multiple solid tumors applied Poly ICLC intratumorally (i.t.) or intramuscularly (i.m.), showing good tolerability with local and systemic immune responses, achieving clinical benefit (188). Two trials investigating melanoma have also been completed with available results: in phase I/II trial NCT01079741, poly ICLC was combined with NY-ESO-1 protein vaccine and Montanide to treat melanoma, effectively activating humoral and T cell immunity in patients. In phase I trial NCT01585350, poly ICLC together with peptide vaccine and incomplete Freund’s adjuvant (IFA) markedly enhanced T cell responses (190). Phase I trial NCT01834248 also combined poly ICLC with NY-ESO-1 vaccine and Decitabine in treating myelodysplastic syndrome, which showed success in the induction of NY-ESO-1-specific cytotoxic CD4^+^ and CD8^+^ T cells as well as CD141^Hi^ cDCs (265). The completed phase I trial NCT03380871 combined Hiltonol with a tumor vaccine, pembrolizumab, and chemotherapy to treat NSCLC, which showed a good safety profile and immune stimulation. Two completed phase I trials investigating either Hiltonol combined with tumor vaccines (NCT02721043) or Hiltonol with a tumor vaccine and Nivolumab (NCT02897765), all showing good treatment safety profile and immunogenicity. In particular, the latter treatment strategy induced intra-tumoral chemotaxis of cytotoxic T cells (191). The phase I trial NCT02544880 recently completed, where Hiltonol and Mucin1 were combined to make the MUC1 vaccine; together with influenza vaccine and PDE5 inhibitor Tadalafil, MUC1 vaccine has been used to treat solid tumors. Published interim results showed that the combination of MUC1 vaccine and Tadalafil was well tolerated and induced immune activation in HNSCC patients, despite the implication of immune evasion after the treatment was also observed (192). However, trial NCT01532960, where Hiltonol was combined with a tumor vaccine in treating breast cancer, was terminated due to the lack of immune stimulation by of Hiltonol for the vaccine (266). Phase II trial NCT02873819, phase I/II trial NCT02643303, and phase I trial NCT03358719 usingHiltonol in combinatory cancer therapies have recently closed, and are awaiting results. In general, Hitonol is well tolerated and can induce immune responses; however, there is a lack of robustdata presenting its competence in bringing clinical benefit.

Rintatolimod is a modified poly (I:C), PolyI: PolyC12U, administratedintravenously (i.v.) or intraperitoneally (i.p.) in gynecological cancers, breast cancer, and colorectal carcinoma. In clinical trials, Rintatolimod is applied used in combination with chemotherapy, a tumor vaccine, ICB, or other immune-modulatory therapies. Phase I/II trial NCT01312389 and phase II trial NCT03403634 using Rintatolimod have been completed, however the results are not yet publicly available.

BO-112 is a nanoplexed form of poly (I:C), which is currently under investigation in melanoma with pembrolizumab (NCT04570332).

In conclusion, pre-clinical data of TLR3 agonists provide promising perspectives for them to move from bench to bedside, yet more research and trials are needed in optimizing TLR3Ls’ clinical effectiveness.

### TLR 4

In tumor studies, TLR4 has been reported to enhance the function of antigen-presenting cells (APCs) (267), increase the production of pro-inflammatory cytokines as well as IFNs (268–270), and boost cytotoxic responses of CTLs and NK cells (267, 271). These immune stimulating mechanisms can be initiated by TLR4 agonists in cancer treatment.

Recently, lipopolysaccharide, lipid A derivatives, polysaccharides, and protein TLR4 agonists were investigated in therapeutic studies (**Table 1**). These TLR4 agonists have been mostly used in combination with tumor vaccines, including DNA-, peptide- and DC-based vaccines; they have also been used in combinationwith radiotherapy and different kinds of immune-stimulating agents. Notably, many TLR4 agonists are engineered into nanoparticles, which have improved drug delivery efficiency and reduced off-target effects (88–91).

Several TLR4 agonists have completed clinical studies(**Table 2**), including LPS, GLA-SE(G100), IDC-G305, GSK1795091 (CRX-601), and the TriMix DC vaccine.

The completed phase I trial NCT01585350, investigated LPS in combination with a peptide vaccine and incomplete Freund’s adjuvant (IFA) to treat melanoma, which showed that this treatment was well tolerated and stimulated marked T cell responses (190).

GLA-SE is a glucopyranosyl lipid A-stable oil-in-water emulsion, tested in lymphoma, skin cancers, sarcoma, lung cancer, and colorectal cancer, either applied alone or in combination with surgery, chemotherapy, radiotherapy, ICB, or a tumor vaccine. In these trials, GLA-SE is administered i.t. or i.m. A phase I trial NCT02035657 applying GLA-SE in treatment against Merkel cell carcinoma has been completed. Treatment withintratumoral GLA-SE as an adjuvant to surgery and radiotherapy demonstrated safety and feasibility in Merkle cell carcinoma with increased intratumoral infiltration of CD8^+^ and CD4^+^ T cells, activation of immune-related genes, and local tumor regression (193).

IDC-G305 is a combination of NY-ESO-1 recombinant protein and GLA-SE, that has been tested in melanoma, ovarian cancer, renal cell cancer, and non-small cell lung cancer(NSCLC) (NCT02015416). This phase I trial showed that intramuscular administration of IDC-G305 was well-tolerated and can generate antigen-specific immunity (195). On the contrary, another two phase I trials NCT02387125 and NCT02609984, where NY-ESO-1 recombinant protein-GLA-SE was used with a DC-targeting tumor vaccine in treating solid tumors including sarcoma, melanoma, NSCLC, and ovarian cancer, were terminated because of lack of efficacy.

GSK1795091, a synthetic aminoalkyl glucosaminide 4-phosphate, in conjunction with anti-PD-1 pembrolizumab was investigated in advanced solid tumor treatment in trial NCT03447314; the results of this trial are yet to come.

TriMix DCs are autologous DCs incorporating mRNA encoding, CD40 ligand, CD70, and a constitutively active TLR4. The phase I/II trial NCT01530698 showed TriMix DCs, further electroporated with a tumor antigent encoding mRNA, administered intranodally, was safe but with only narrow immunological and clinical response.

In general, recent clinical trials usingTLR4 agonists did show clinical or immune stimulating efficacy in solid tumor treatment. However, there are contradictory results about the same agent in different trials. For those agents that showed promising results in early phase trials, future studies should pay attention to the optimal treatment conditions, including the therapy combination strategy and the route of administration.

### TLR 5

TLR5 can be activated by bacterial flagellin and plays a key role in the immune homeostasis of pathogen-host interactions (272, 273). In addition, TLR5 is associated with decreased tumor cell proliferation, invasion, and metastasis (274, 275). Several drugs targeting TLR5 are under investigation.

Recently, protein-based TLR5 agonists have been investigated pre-clinically: in combination with a tumor vaccine (96), incorporated into the adoptive cell therapy (97), or used alone (98). All yielded enhanced therapeutic effects (**Table 1**).

Entolimod and Mobilan are administrated in two phase I trials (**Table 2**). Entolimod, namely, CBLB502 is a derivative of Salmonella flagellin. In trial NCT01527136, i.m. or s.c. administration of Entolimod was usedto treat locally advanced or metastatic solid tumors that cannot be removed by surgery. Results of this trial are yet to be available. While having the advantage of avoiding the dangerous inflammatory “cytokine storm” (276), Entolimod has a drawback that it can induce a rapid antibody neutralization (277),leading to its efficacy limitation. Pharmacological modification could be one solution to this problem. Mett et al. (277) identified and eliminated the epitopes leading to Entolimod’s neutralizing immunogenicity and refined its structure to get GP532 as a new TLR5 agonist. GP532 reduced neutralizing antibody response while preserving pro-inflammatory capacity. However, the anti-tumor potential of GP532 has not been verified in any therapeutic studies yet.

To extend the application of TLR5 agonism in TLR5 negative tumors, an adenovirus carrying TLR5 receptor and its agonist’s gene called Mobilan (M-VM3) was designed (276). In preclinical prostate cancer models, Mobilan displayed promising therapeutic efficacy (276).In phase I clinical trial NCT02654938, Mobilan was used intratumorally to treat prostate cancer; no results are available yet. A similar study showed Mobilan was well-tolerated in prostate cancer patients (278); however, more data is needed to verify its clinical benefit.

### TLR 7 and TLR 8

TLR 7 and 8 serve as important sentinels in response to infection, which makes them irreplaceable in the activation of mammalian innate immune cells (279). These two receptors have also been found to participate in the regulation of the host adaptive immunity (280–283). The emergence of novel small molecular agents targeting these two receptors in the last decade has greatly facilitated cancer immunotherapy studies by adopting their immune activation potential.

Since many agonists exert dual activation to both TLR7 and TLR8, we will summarize TLR7 and TLR8 agonists together in one section.

Following pre-clinical investigations TLR7 or TLR7/8 agonists can be classified by molecular nature into imidazoquinoline and its derivatives, novel synthetic small molecules, and RNA-based agents (**Table 1**). These agonists are studied either with traditional therapies, such as chemotherapy, radiotherapy, surgery, or novel therapies, such as tumor vaccines, immune checkpoint blockade, adoptive cell therapy, and photothermal therapy (**Table 1**). It is noteworthy that nanotechnology has become a hot research topic in the drug delivery of therapies applying TLR7/8 agonists. TLR7/8 agonists are incorporated into multi-functional nanoparticles that overcome drug solubility limitations (120, 122) or improve tumor-specific environment-targeting ability (115, 124, 125), thus improving drug delivery efficiency.

A number of TLR7 agonists that have undergone clinical investigation include imiquimod, TMX-101, RNA-LPX, BNT411, DSP-0509, LHC165, SHR2150, TQ-A3334, and RO7119929 (**Tables 2** and **3**).

Imiquimod has already been clinically applied in the treatment for genital warts (284), low-risk superficial basal cell carcinoma (285), and Bowen’s disease (cutaneous squamous cell carcinoma in situ) (286).In clinical trials initiated in the decade, Imiquimod has been utilized in a wide range of clinical trials targeting different tumor types. There are studies attempting to widen the utilization of Imiquimod based on the current standard of care.

Many clinical studies of Imiquimod focus on Skin cancer. In basal cell carcinoma, a phase III trial (NCT05212246) is going to use Imiquimod as a single treatment in high risk populations to prevent tumor occurrence; imiquimod is also applied in two active trials in combination with curettage (NCT02242929, phase III), and sonidegib together with surgery (NCT03534947, phase II) to treat basal cell carcinoma. In an active phase I trial (NCT03370406), Imiquimod together with 5-fluorouracil is applied to treat squamous cell carcinoma. Two active phase I trial are investigating melanoma, where Imiquimod is combined with either Pembrolizumab(NCT03276832) or tumor vaccine(NCT04072900). A completed phase III trial NCT02385188 found imiquimod together with paracetamol and lidocaine induced 82.6% response rate in vulvar Paget disease but 80% of patients needed painkiller to mitigate side effects (197).

Imiquimod is also studied in intraepithelial neoplasia. In grade 2/3 cervical intraepithelial neoplasia (CIN), a phase II/III trial (NCT02130323) showed that Imiquimod induced lower HPV clearance rate compared to large loop excision (43% vs 64%), which made conization remain the standard care when intervention is needed (199).However, trial NCT03233412 showed that when adjuvant to the loop excision procedure, weekly application of imiquimod can promote regression of high-grade squamous intraepithelial lesions (200). Two active trials are investigating Imiquimod combining HPV vaccine (phase II, NCT02864147) or topical Fluorouracil (phase I, NCT03196180) in treating CIN2/3. In vaginal intraepithelial neoplasia (VIN), the results of the phase III trial NCT01861535demonstrated that: Imiquimod is safe and effective in treating VIN and should be considered as a first-line treatment alternative to surgery (198).In the active phase III trial NCT02059499, single use of Imiquimod is applied in HIV patients who have anal intraepithelial neoplasia.

In glioma, results of phase I trial NCT02454634 recently published in *Nature* (202) showed that topical Imiquimod combined with IDH1 peptide vaccine induced 93.3% immune responses in IDH1R132H-mutated patients while adverse effects are within grade I. The immune responses induced by the treatment were represented by enhanced IL-17 production and increased IDH1-specific T cells (202). Trial NCT00899574 focused on breast carcinoma; the results showed that single topical use of imiquimod proved beneficial for breast cancer metastasis to skin/chest wall and has been well-tolerated; additionally, this trial proved that imiquimod could induced a pro-immunogenic TME (201). There are also completed phase I/II trials using Imiquimod with radiotherapy, chemotherapy or tumor vaccine to treat breast cancer (NCT01421017, NCT02276300).

In prostate cancer, Imiquimod is applied topically with different tumor vaccines (NCT02293707, NCT02234921). The phase II trial NCT02293707 showed the combination of Imiquimod, telomerase peptide-based vaccine, and Montanide ISA-51 VG was safe in prostate cancer patients. The number of treatments was positively associated with immunological response (204). Bladder cancer is another type of malignancy where Imiquimod displayed its therapeutic potential. TMX-101 is the intravesical formulation for Imiquimod; single use of TMX-101 induced significant increase in urinary cytokines, including IL-6, IL-18, IL-1β, IL-1 receptor antagonist (IL-1ra), and vascular endothelial growth factor (VEGF), in non-muscle invasive bladder cancer, despite accompanied with mild adverse effects (phase II, NCT01731652) (205).

In general, Imiquimod showedits potential in cancer treatment, and also an increasing number of active trials using it has been initiated.

A trial with RNA-LPX (NCT02410733) showed early results with effective quick induction of IFNα and IP-10, and the induction of *de novo* antigen-specific T-cell responses. In this study, i.v. administration of RNA-LPX proved to be well-tolerated (116). SHR2150 and RO7119929 are oral TLR7 agonists, currently in trials in combination with other therapies in metastatic solid tumors (SHR2150, NCT04588324), and liver/biliary tract cancer (RO7119929, NCT04338685). BNT411 and DSP-0509 are synthetic small molecular TLR7 agonists administered i.v. that are being investigated in multiple types of tumors. LHC165 is a benzonapthyridine TLR7 agonist, which has been used in combination with anti-PD-1 (PDR001) to treat solid tumors.

TLR8 agonist VTX-2337, also known as Motolimod, has been tested in ovarian cancer and squamous cell cancer patients combined with chemotherapy, ICB, or small molecular inhibitors; in most studies, Motolimod was applied subcutaneously. Four studies have since completed with published results. In the first trial, a phase I trial (NCT01294293) with different histological subtypes of ovarian cancer, Motolimod was combined with doxorubicin, showing 15% patients with a complete response and 53% with stable disease (210). A similar phase II trial (NCT01666444), however, showed that the supplement of Motolimod to doxorubicin did not improve clinical outcomes in patients with recurrent or persistent epithelial ovarian, fallopian tube or primary peritoneal cancer, compared with placebo (207). Motolimod was demonstrated to augment clinical responses in patients with HNSCC of advanced stages, who have also been treated with the epidermal growth factor receptor inhibitor cetuximab (phase I trial NCT01334177) (209). However, in a trial of recurrent or metastatic SCCHN patients, the addition of Motolimod to platinum-based chemotherapy and cetuximab did not improve progression free survival (PFS) or OS; nevertheless, remarkable benefit was presented in HPV-positive patients and those with injection site reaction, suggesting the application potential of Motolimod in subset- and biomarker-selected patients (phase II trial NCT01836029) (287). Recently, a strong TLR8 agonist, combined with a HER2 monoclonal antibody was designed (sbt-6050), aiming to activate myeloid cells with the presence of HER2 positive tumor cells, and it is under investigation now in HER2 positive solid tumors with pembrolizumab (NCT04460456).

In terms of dual TLR7/8 agonists, Resiquimod, TransCon, 852A, BDB001, BDB018, NKTR-262, and BDC-1001 have undergone clinical investigation. Resiquimod has been examined by topical use in melanoma and brain tumors in combination treatment with a tumor vaccine and/or other adjuvants, however the addition of Resiquimod to NY-ESO-1 protein vaccine and IFA failed to elicit steady antigen-specific CD8+ T cell response (NCT00821652) (206). TransCon is a type of Resiquimod prodrug, now under investigation in combination with pembrolizumab in solid tumors (NCT04799054). 852A is also an imidazoquinoline TLR7/8 agonist, which has previously been tested in multiple tumors types (including renal cell carcinoma, melanoma, lung cancer, hematologic malignancies, breast cancer, ovarian cancer, and cervical cancer), showing acceptable tolerability, evident immune stimulation, and clinical benefit in some patients (288–291). The i.v. administered TLR7/8 agonist BDB001 and its refined analog BDB018 (designed to enhance immune stimulation while preserving the safety profile of BDB001) are both investigated in solid tumor treatment (NCT03486301, NCT04196530, NCT04840394). BDC-1001 is an immune stimulating antibody conjugate of an anti-HER2 monoclonal antibody with a TLF7/8 dual agonist, currently recruiting a trial enrolling patients with HER2 positive solid tumors (NCT04278144).

In summary, TLR7/8 agonists have drawn most research attention among other TLR ligands; despite that preclinical data indicate promising prospects, none of the TLR7/8 agonists has gained regulatory approval to treat cancer in human except Imiquimod. Future study could focus on optimizing delivery routes, administration schedule, and the vaccine itself (where vaccines are applied) (292).

### TLR 9

TLR9 is preferentially expressed on the endosomal membrane of B cells and plasmacytoid dendritic cells (pDCs), and its primary ligand is unmethylated cytidine phosphoguanosine (CpG) oligonucleotides (ODNs) (293). TLR9 activation can lead to potent activation of innate (133, 294, 295) and adaptive immunity (both cell and humoral immunity) (133).

TLR9 agonists previously evaluated in pre-clinical studies are all DNA-based agents containing non-methylated CpG motifs (**Table 1**). Most of these agonists are CpG ODNs, while some have more complex DNA structures designed for better protection from drug degradation and enhanced TLR9 stimulation capacity (164–166). The therapeutic enhancing effect of TLR9 agonists has been studied with chemotherapy, photodynamic therapy, radiofrequency ablation, vaccines, ICB, and other immune stimulatory agents. (**Table 1**) Interestingly, in two studies (139, 140) the CpG motif has been used as myeloid cell targeting sequence, incorporated into decoy ODNs that contain STAT3-specific sequence, which can inhibit STAT3 transcription. Both studies showed the decoy ODNs effectively suppressed tumors in animal models of hematological malignancies. Similar to TLR7/8 agonists, many studies have also looked into the design of nanoparticles for TLR9 agonist drug delivery. These versatile nanoparticles have improved the ability for TME-targeting (159), specific cell-targeting (148, 150, 163), and even sub-cellular compartment-targeting (147); they have also protected against drug degradation (152, 156); plus, some conjugated to drug tracers to monitor drug tracking after administration *in vivo* (152, 155).

Currently the most clinically investigated TLR9 agonists (**Tables 2**, **3**) are SD-101, CMP-001, IMO-2125. SD-101 is a class C CpG oligonucleotide, applied intratumorally in trials for advanced (metastatic, refractory, or recurrent) malignancies such as pancreatic adenocarcinoma, prostate cancer, liver metastatic uveal melanoma, and multiple lymphoma subtypes. In these early phase trials, SD-101 will be administered in combination with ICB, radiotherapy, small molecular inhibitors, or an anti-OX40 antibody.

Two phase I/II trials applying SD-101 in combination with radiotherapy and immunotherapy in treating lymphoma and other solid tumors just completed, and the results are not yet available. CMP-001, also named as Vidutolimod, is a short DNA piece packaged in protein, mimicking the virus structure. It has been administratedsubcutaneously or intratumorally with ICB, an OX40 agonist, an IgG2 agonist, surgery, and radiotherapy in multiple types of primary and metastatic solid tumors. The completed phase I trial NCT03084640 is completed, which combinedPembrolizumab with CMP-001, and compared the safety together with response rates of CMP-001 administered in different routes in intreating melanoma. Another completed phase I trial NCT03438318 combined CMP-001 with Atezolizumab and radiotherapy to treat NSCLC. In both of the above trials, CMP-001 elicited antitumor-related transcriptional signatures (2022 AACR Abstract #: LB107); more results from trial on CMP-001 are yet to be published. IMO-2125, also known as Tilsotolimod, a synthetic phosphorothioate oligodeoxyribonucleotides, has shown a good safety profile with effective activation of dendritic cells, induction of type I IFN signaling, and up-regulation of multiple immune checkpoint pathways in a phase I trial (NCT03052205) (213), where IMO-2125 was usedas a monotherapy administrated intratumorally in refractory solid tumors. However, a phase III trial (NCT03445533), where IMO-2125 and Ipilimumab were combined for treating melanoma, was terminated for lack of efficacy. Other phase I to II trials with intratumoral administration of IMO-2125 are actively recruiting, either alone or with the combination of ICB in solid tumors (**Table 3**).

MGN1703 (Lefitolimod) is a DNA-based molecular alternative to a CpG-ODN, administratedsubcutaneously in trials and currently under investigation in combination with anti-CTLA4 in advanced cancers (NCT02668770). In trial NCT02200081, MGN1703 was used with platinum-based chemotherapy in advanced stage small-cell lung cancer where it was well tolerated but showed no impact on the main efficacy of treatment and overall survival of patients (214). DUK-CPG-001 a CpG-based TLR9 agonist is being evaluated in the treatment of myeloid and lymphoid malignancies combined with donor lymphocyte infusion enriched with NK cells in a phase II trial (NCT02452697).

Other TLR9 agonists, all CpG-based, have been assessed in clinical trials, with results available. Inhaled DV281 was tested with anti-PD-1 in advanced NSCLC, with no results publicly available yet (NCT03326752). CpG-MCL vaccine, combining CpG7909 and autologous mantle cell lymphoma-derived tumor cells, was studied to treat mantle cell lymphoma in combination with immunochemotherapy and an autologous hematopoietic stem cell transplant (NCT00490529). The trial showed moderate response to the vaccine among patients (40% developed anti-tumor CD8 T cell response, associated with favorable clinical outcomes), good treatment efficacy (89% were minimal residual disease negative), and a good safety profile (211). CpG7910 was applied in combinationwith local radiotherapy to treat recurrent low-grade lymphoma in a phase I/II trial (NCT00185965), where local administration of this agent systemically induced tumor-reactive memory CD8^+^ T cells. This regimen showed clinical feasibility (212). EMD 1201081 combined with EGFR inhibitor, however, showed no improvement to the clinical efficacy of HNSCC treatment (215).

In summary, despite that there are trials showing the immunogenicity and good tolerability of CpG-based agents, low translational rate remains an issue. This may due to the difference in TLR9 expression in animal models and humans (296). Pre-clinical models of human origins are necessary, such as primary tumor organoids. Also, one safety concern is that CpG motifs are potential to induce autoreactivity (297). There need to be trials with larger sample sizes, longer follow-up durations, and stratified patient subgroups to verify the safety and the suitable patient groups that can benefit from CpG ODN adjuvanted therapies.

### Multi-TLR

In addition to single TLR activation, multi-TLR agonism is also under both pre-clinical and clinical investigation for many cancers. Multi-TLR agonism can be realized by a single agent or by a combination of several TLR agonists.

Preclinically, the most studied treatment modality with multi-TLR activation is with tumor vaccines (**Table 1**). There is also a trend of applying nanotechnology in drug delivery in multi-TLR stimulating treatments for improved APC-targeting (173, 179) and drug tracing (170).

In clinical trials, Bacillus Calmette-Guérin (BCG), an attenuated live Mycobacterium bovis, is the most studied multi-TLR agonist. It can activate TLR2, TLR4, and TLR9 (298); it was originally applied as a vaccine against tuberculosis and later broadened its application as an adjuvant in the standard care of a part of bladder cancer patients (298). Currently, BCG is still investigated in bladder carcinoma patients in combination with other drug treatments, including an anti-PD-1/PD-L1 antibody, small molecule inhibitors, and a neoantigen encoding gene vaccine. A completed trial NCT02753309 combined rapamycin with BCG in bladder cancer treatment, which showed good tolerability in high-grade non-muscle invasive bladder cancer patients and induction of antigen-specific γδ T cell response as well as urinary cytokine production (219). BCG has also been assessed for the treatment of upper or lower urinary tract carcinoma. In treating urinary tract urothelial carcinoma, the trial NCT00794950 investigated combination therapy of BCG and sunitinib, a novel anti-angiogenesis drug. This trial has since been completed, and results showed the combination treatment was associated with a lower rate of progression and recurrence with an acceptable safety profile (218). Besides malignancies in the urinary system, BCG combinedwith chemotherapy, radiofrequency ablation, and GM-CSF have been evaluated in the treatment of liver metastatic colorectal cancer (NCT04062721).

OM-174 is a lipid A analog, which activates both TLR2 and TLR4. In a phase I trial (NCT01800812) with solid tumor patients, OM-174 as a monotherapy was well tolerated and proved to be effective for the induction of IL-8, IL-10, TNF-α, and IL-6 (despite that TNF-αand IL-6 decreased progressively in repeated treatment). Progressively increased NK cells and NK cell activity were observed in patients who received the highest dose, 1000 μg/m^2^ (217). AS-15 is an adjuvant system, comprised of a TLR4 agonism system (MPL and QS-21) and a TLR9 agonist (CpG 7909) in a liposomal formulation. In the completed phase II trial NCT01266603, AS-15 was combined with the MAGE-A3 tumor antigen and high dose IL-2 to treat metastatic melanoma. This treatment achieved 25% response rate, which was associated with increased infiltrating T cells in the primary tumor tissue (222).

TLR3 agonist poly-ICIC and TLR7/8 agonist Resiquimod have been combined with tumor vaccines in the treatment of melanoma (NCT02126579) and advanced tumors refractory to conventional treatment (NCT00948961). In the completed trial NCT00948961, poly-ICIC and/or Resiquimod combined with NY-ESO-1-targeting DC cell vaccine. The combination therapy induced NY-ESO-1–specific IgG titers in 79% patients and NY-ESO-1–specific T cell responses in 56% patients, leading to disease stabilization and tumor regression in 15/70 patients (220).

A phase I trial NCT00068510, where poly-ICLC or imiquimod used in conjunction with an autologous tumor-pulsed DC vaccine, has reported that this regimen was safe and effective in prolonging survival for glioblastoma patients (221).

### TLR antagonists

Considering the versatility of TLRs in cancer, some of which can promote tumor growth, there is also rationale for exploring TLR antagonists as a cancer therapy.

In several types of cancers arising from the digestive system, pre-clinical studies have demonstrated the therapeutic potential of TLR antagonists. OPN-301, an inhibitory anti-TLR2 antibody, suppressed tumor initiation and growth in a gastric animal model, which was associated with gene suppression of CXCL2 and TNF-α (183). Another two studies reported that IRS-954, an inhibitory DNA sequence against TLR7 and 9, and chloroquine, which inhibits TLR7 and 9 activation, significantly impeded tumor development and growth *in vivo* and *in vitro* in liver cancer (184) and cholangiocarcinoma models (185).

A subset of diffuse large B cell lymphoma patients carry the MyD88 L265P mutation, associated with overactivation of the TLR7/9/MyD88 pathway (186). A TLR7/8/9 antagonist IMO-8400 was previously usedin this subset of lymphoma patients in a completed clinical trial NCT02252146, which, showed limited therapeutic efficacy. Similarly, trial NCT02092909, studying IMO-8400 in treating Waldenstrom’s macroglobulinemia, also showed insufficient efficacy of IMO-8400, and hence it was terminated.

Recently, another synthesized ODN-based TLR7/9 antagonist HJ901 was shown to significantly inhibit tumor cell proliferation and tumor growth in cell lines or animal models carrying the MyD88 L265P mutation (186). However, these data warrant further investigation before it can be translated to the establishment of an early phase trial.

There are obviously fewer published studies on anti-cancer TLR antagonists, which generally showed less efficacy than TLR agonists.

## TLR-targeted therapy: Challenges and future perspectives

### Simultaneous activation of anti-/pro-tumor mechanisms

As TLRs regulate a wide range of functions, initiating signaling pathways that may lead to both anti-tumor and pro-tumor effects, there is a concern regarding whether the application of TLR agonists will promote tumor growth or whether the anti-tumor effect will be counteracted by the pro-tumor effect they simultaneously initiated. For example, Theodoraki and colleagues have found that TLR3 agonists stimulate the TLR3-TRAF3/IRF3 pathway, which leads to the production of CTL attractants and activate MAVS/helicase pathway, which elicits Treg chemotaxis (299).

**Figure 3:**
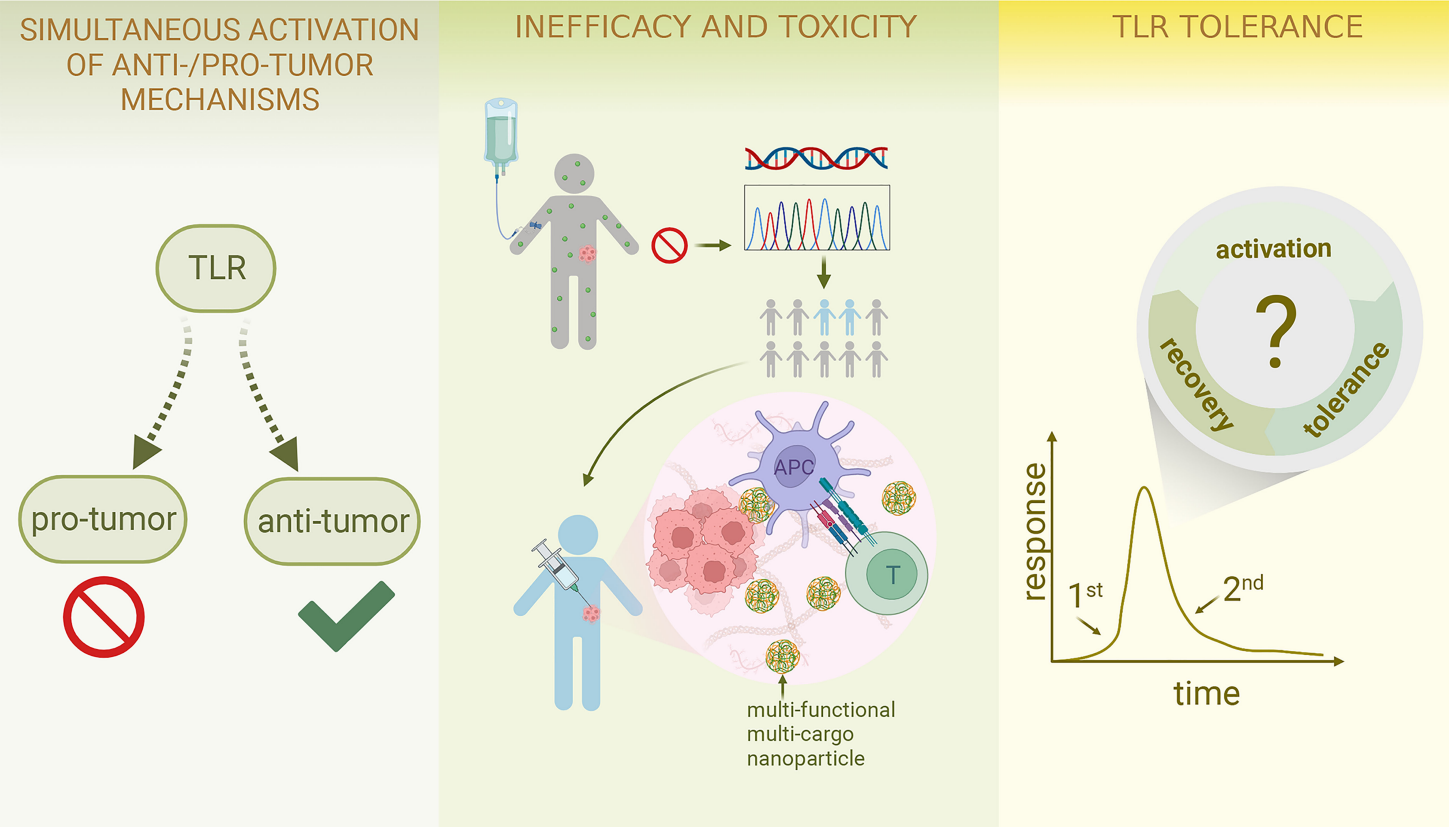
Challenges and Future Perspective of TLR-targeting tumor therapies Due to the versatile nature of TLRs, simultaneous activation of both anti-tumor and pro-tumour effects can arise; future work may involve combination therapy and further exploration of the association between ligand conformation and specific pathway activation as well as the development of pathway-specific drugs. To resolve the inefficacy and toxicity of TLR-targeting drugs, identifying an appropriate patient population is of great significance; combination therapy again can be considered; systemic drug administration can shift to localized delivery; the incorporation of polymer and nanotechnology is an emerging field. TLR tolerance could be a major obstacle preventing the TLR-targeting therapies from achieving their full potential; thus, more effort should be put into studying cyclical changes in TLR-TLRL interaction and investigating the optimal treatment dose and schedule. (This figure is created with BioRender.com).

Two directions may be feasible for further investigation into this challenge. On one hand, choosing the agonist that specifically activates the anti-tumor pathways but leaves no effect on the pro-tumor pathway could help (**Figure 3**). Theodoraki et al. (299) further discovered the difference of pathway stimulation potential in different TLR3 agonists: dsRNA Sendai Virus, Poly (I:C), and Rintatolimod. Rintatolimod can activate the TLR3 pathway only, resulting in the induction of CTL attracting cytokines IFNa, ISG-60, and CXCL10, and avoid the initiation of MAVS/helicase pathway, which then prevents Treg accumulation. In the future, more focus to elucidate how drug conformation of TLR-targeting agents influences downstream signaling is necessary, which can then guide future drug synthesis or discovery.

On the other hand, combination therapy is of utmost significance in TLR-targeted therapy. Combined therapies can enhance therapeutic effects in many cases and potentially diminish the pro-tumor impact elicited by TLR agonists. Feng et al. (300) discovered that poly (I:C) could inhibit HCC cell proliferation and lead to cell apoptosis while promoting cell migration and invasion at the same time. When combined with the toad venom constituent bufalin, cell migration and invasion were inhibited, whereas cell apoptosis and suppressed proliferation remained unaffected (300), indicating the potential of combination therapy of the two in eliminating poly (I:C)-initiated pro-tumor effect.

### Inefficacy and toxicity

Despite numerous promising results emerging from pre-clinical research with TLR-targeted therapies, many failed to advance to clinical implementation. Lack of efficacy and toxicity has been an issue for TLR-targeting therapy (28, 301).

To address this, first, precise identification and investigation of patient sub-populations that respond to TLR-targeting strategies is important (**Figure 3**). Some treatments may not have shown significant efficacy in the overall study population, but do in a subset of patients. Monk et al. (207) and Ferris et al. (208) both found in their clinical trial studies that adding Motolimod to chemotherapy did not improve survival in the overall patient population. In contrast, the sub-population with injection site reactions did significantly benefit from Motolimod. The evidence above suggests 1) lack of significance in the overall analysis does not reject the potential of a certain TLR-targeting drug; 2) there are certain groups of patients that may be more responsive to TLR-targeting agents than others. Future work calls for more translational studies incorporated into the trial designs to examine any associations between treatment responsiveness and patient characteristics, including gene expression patterns, biomarker levels, local treatment reaction, etc., thus providing clues for the screening of the target patient population.

Secondly, both lab and clinical research data support that the optimal treatment potency of immunotherapy requires combinatory approaches (27, 28), which continues to be the trend for TLR-targeted therapy. As discussed in section 5 (**Tables 1**–**3**), many studies showed that the combination of TLR agonists with other therapies enhanced tumor retardation compared to a single treatment alone. However, there is still a gap in our knowledge regarding the timing and dosing to be applied to each treatment to achieve the optimal therapeutic result. For example, in the combinatory treatment of poly (I:C) and radiation in Lewis lung carcinoma models, Yoshida et al. (264) found using poly (I:C) one day ahead of radiotherapy yielded better tumor suppression than to use after radiation.

Pharmaceutical modification of targeting agents has great potential to improve efficacy and lower toxicity as well. Furthermore, polymer and nanoparticle formulations incorporating TLR agonists are promising avenues to explore as they may potentially increase the specificity of targeting and prevent the drug from early degradation, thereby reducing systemic toxicity. Those formulations can now be designed to load multiple agents and be multi-functional (**Figure 3**), including but not limited to pH-specific drug-releasing (115, 124), *in vivo* drug tracing (152, 155), and tumor hypoxia relief (106). The advancement of nanotechnology utilizedin TLR-targeted therapy has been reviewed comprehensively elsewhere (302). However, careful attention should be paid to these new biomaterials as the bioactivities they may incur *in vivo* remain to be fully elucidated. Meanwhile, reducing the financial and timing costs is also a formidable challenge to face.

Besides refinement of the drug delivery system, choosing a localized delivery route may also have an influence on efficacy. This can avoid systemic inflammation to a great extent and improve intratumoral efficacy (300), especially for solid tumors.

### TLR Tolerance

TLR tolerance arises due to the unresponsiveness or hyporesponsiveness of TLRs upon TLR agonist stimulation after repeated, prolonged, or chronic activation, which also includes TLR cross-tolerance, where a pre-used TLR agonist incurs tolerance of another TLR (303, 304). There have been studies showing TLR tolerance in TLR2, 3, 4, 5, 7/8, and 9 (304–309). This mechanism physiologically prevents uncontrolled inflammation and autoimmunity, thus protecting healthy tissues from inflammatory damage (303, 304). In cancer, however, such tolerance leads to impaired tumoricidal effect.

To overcome this phenomenon, Bourquin et al. (310) found cycles of repeated low dose TLR7/8 agonist injection within 24h with intervals every five days, compared to one high dose injection every three days, significantly suppressed tumor growth in a mouse model (310), indicating the potential of circumventing TLR tolerance by adjusting treatment dose and time. Similarly, Tsitoura et al. (311) found the minimum and optimal dosing interval to maintain a TLR agonists’ pharmacological responsiveness. Future research needs to further explore the cyclical changes in specific TLR-TLRL induced responsiveness and consider cross-tolerance as new cancer therapies incorporating multi-TLR agonism continue to emerge (**Figure 3**).

## Discussion

The pattern recognition receptor family of TLRs, widely expressed on both healthy cells and tumor cells, play versatile roles in both physical and pathological conditions. Each TLR influences multiple aspects of tumor development, with both anti- and pro-tumor potentials.

TLR-targeted cancer therapies have been widely studied, with some TLR agonists (Imiquimod and BCG) approved for clinical use to treat cancers (312). Pre-clinical studies are currently focusing on developing novel TLR-targeting agents, incorporating nanotechnology into TLR drug manufacturing and delivery, and novel approaches activating TLRs apart from using agonists (e.g. inactivated virus (67), engineered bacteria (175), and modified T cells (97)).(**Figure 2**) Increasing evidence demonstrates the insufficiency of single intervention in circumventing immunosuppression incurred by tumor progression, hence leading to both pre-clinical and clinical research focusing on multi-agent and multi-modality treatment at present (312). (**Figure 2**) In addition, TLR antagonists also showed therapeutic potential in pre-clinical models with tumors in the digestive system, but the potency of this group of agents in human remains to be fully elucidated.

Future research into TLR-targeting therapies must tackle several challenges, including the control of simultaneous activation of both anti- and pro-tumor effect elicited by TLR activation, TLR tolerance, and insufficient efficacy and toxicity. Additionally, due to the heterogenous nature of different types of tumors and the consequent influence on TLR activities in the TME, better research models, which preserve the important genetic mutations that affect tumor growth and treatment response, should be developed (313). Instead of using cell lines, bulk tumors derived from the real patient could be an ideal source for research models (252), from where the primary TME cell components– tumor cells, stromal cells, and immune cells–can be extracted.

Furthermore, with the trend of precision medicine and the deepening of research, there is a need for patient stratification according to their disease profile and treatment responsiveness to treat each patient with the most suitable strategy, providing them with the optimal benefit both therapeutically and economically. We speculate that addressing the challenges described above will elevateTLR-targeted therapies to their full potential and augment the efficacy of immunotherapies for patients.

## Author contributions

YY and HL drafted the manuscript and organized the tables. YY designed the figures. XZ raised the article topic and designed the original manuscript structure. CF and PC modified the manuscript and proofread the language. All authors contributed to the article and approved the submitted version.

## Funding

This work was supported by the National Natural Science Foundation of China (No. 81821002), National Natural Science Foundation of China (No.81902662), and Sichuan Science and Technology Program 2021YJ0011.

## Conflict of interest

The authors declare that the research was conducted in the absence of any commercial or financial relationships that could be construed as a potential conflict of interest.

## Publisher’s note

All claims expressed in this article are solely those of the authors and do not necessarily represent those of their affiliated organizations, or those of the publisher, the editors and the reviewers. Any product that may be evaluated in this article, or claim that may be made by its manufacturer, is not guaranteed or endorsed by the publisher.
